# Supplementary material for: Effects of combining sensory-motor exercises with transcranial direct current stimulation on cortical processing and clinical symptoms in patients with lumbosacral radiculopathy: An exploratory randomized controlled trial
Source: PLoS One. 2024 Dec 19;19(12):e0314361. doi: 10.1371/journal.pone.0314361 (PMC11658635; doi:10.1371/journal.pone.0314361)
Supplement: S1 File — (DOCX) [file pone.0314361.s002.docx]

**Effects of combining sensory-motor exercises with transcranial direct current stimulation on cortical processing and clinical symptoms in patients with chronic low back pain: A sensory and motor-evoked potential study**

**Soheila Qanbari**, MSc^1^, **Roya Khanmohammadi**, Assistant Professor, PhD^1^*, **Gholamreza Olyaei**, Full Professor, PhD^1^, **Zohreh Hosseini**, MSc^1^, **Hanie Sadat Hejazi**, MSc^1^.

1. Physical Therapy Department, Tehran University of Medical Sciences, Tehran, Iran.

**1. Introduction and Problem Statement**

Currently, low back pain is one of the most common problems worldwide, with a prevalence rate of 80 to 85%, leading to disability throughout a person's life (1, 2). Among these individuals, 5 to 10% progress to chronic low back pain, which results in high treatment costs and significant pain and suffering (3). Low back pain has led to a restriction of approximately 39% of activities globally (4).

In general, chronic low back pain is defined as pain that persists for more than 3 to 6 months (5). Chronic pain is a complex syndrome that includes emotional, psychological, and mechanical components (6). Despite advancements in diagnostic tools and techniques, determining the exact cause of symptoms is often impossible. What is evident, however, is that in chronic conditions, treatment based solely on correcting structural abnormalities in the musculoskeletal system is often unsuccessful, indicating the presence of other pathophysiological and biopsychosocial mechanisms in chronic low back pain (1, 7).

For over 20 years, increasing research has shown that individuals with low back pain may have alterations in their central nervous system (8). Many pieces of evidence suggest that structural changes in the musculoskeletal system of the lower back may not be the sole contributors and that extensive reorganization in the cortex, such as neurochemical, functional, and structural disorders in the brain, could play a role in the persistence of low back pain. These changes need to be considered from a therapeutic perspective as well. In other words, addressing the pathophysiological mechanisms of chronic low back pain helps in identifying the most effective treatment methods for this group of individuals (7). Recent studies suggest that structural and functional changes in the central nervous system (9) of individuals with musculoskeletal pain reflect adaptive neurophysiological processes and contribute to repair processes by protecting structures. Laboratory and clinical findings indicate that these neurophysiological adaptations might become enduring and progressive, leaving chronic symptoms behind (10).

Indeed, this maladaptive plasticity in the central nervous system in response to pain is one of the most critical phenomena explaining why individuals with chronic low back pain respond poorly to traditional treatments (11). Neuroplasticity is a fundamental and intrinsic neurophysiological process involving structural, functional, and organizational changes in the brain and nervous system that continuously occur throughout an individual's life (10). The primary mechanism of neuroplasticity in the central nervous system involves functional or interference changes in the inherent properties of neurons as well as structural changes in the number or location of synapses between neurons (12). Plastic adaptation initially stems from musculoskeletal structural damage and results in experiencing pathophysiological conditions even without anatomical/structural musculoskeletal damage in chronic conditions (10). Synaptic plasticity involves enduring changes in synaptic quality due to synaptic strengthening or weakening (13).

Evidence indicates that pain is related to maladaptive plasticity in the sensory and motor cortices of the central nervous system (14, 15). These findings complement other behavioral evidence suggesting that there are issues in the sensory-motor function of the lower back in patients, which can ultimately lead to disability (1). Sensory-motor control of the lower back is considered one of the key factors in the progression and recurrence of low back pain (16).

Optimal sensory-motor behaviors depend on precise central processing and integration of sensory information into motor commands (16). There is a complex relationship between perception and action (1), with motor outputs generally depending on sensory inputs. These sensory inputs include somatosensory information about body position and movement and information from the proprioceptive network (8). Sensory information plays a crucial role in selecting the appropriate motor strategy to stabilize and move the spine (1). To maintain spinal posture and control movement, ongoing interaction between motor outputs of the neural pathways related to paraspinal muscles and sensory inputs, including proprioception, at various levels of the nervous system is essential (9, 17).

Overall, pain affects sensory-motor function. When pain occurs, muscle force production capacity decreases; muscular coordination is disrupted (18); body patterns during work change (7); proprioception is impaired (1, 16); tactile accuracy decreases (1, 7, 11, 16); and the ability to integrate sensory information into motor commands is reduced (18). In other words, disruption in sensory-motor control, which encompasses all sensory and motor processing, leading to muscle control and movement as well as maintaining spinal alignment and ultimately ensuring healthy function and appropriate loading of the spine, can be a potential underlying mechanism for the development or persistence of pain (8).

As mentioned earlier, one of the pathophysiological mechanisms proposed for the disruption in sensory-motor control in individuals with low back pain is changes in the sensory-motor processing of the central nervous system. Indeed, findings suggest that in individuals with chronic low back pain, reorganization of brain areas related to sensory information processing may be involved in sensory accuracy related to body perception and play a significant role in the neural control of the lumbar region (1). Overall, reorganization of the sensory-motor system in the brain may cause changes in sensory and motor manifestations, including disruptions in body perception (10) and functional changes in motor planning, ultimately leading to impaired postural control (1).

Regarding sensory processing, studies indicate that in individuals with low back pain (both acute and chronic phases), changes and disruptions occur in the primary sensory cortex (S1), and these changes are strongly associated with pain intensity, functional deficits, and symptom chronicity (11, 19). During acute conditions or experimental muscle pain, decreased excitability in S1 occurs before changes in motor output (19). Additionally, patients with decreased excitability in the S1 region experience more pain compared to those with increased excitability (19). In chronic pain, pain memory progression can alter the somatotopic map of S1 and lead to heightened sensitivity even without pain stimuli (12).

Furthermore, a significant correlation exists between gray matter density in the brain and pain intensity and duration (12). Recent evidence shows that chronic low back pain is associated with reduced gray matter in the somatosensory cortex (20). Other studies have indicated that in chronic low back pain, the thickness of the primary (S1) and secondary (S2) sensory cortices is reduced compared to the control group (11). The S1 region processes unisensory inputs and integrates them with motor signals to guide movement. The S2 region integrates multisensory and bilateral sensory inputs and is a key point in sensory-motor integration through its connections with motor areas (16). S1 also refines motor control (17). Studies have also shown that in individuals with low back pain, the S1 region, which receives information from the trunk, shifts its location in the brain, thereby disrupting its connection with M1 and reducing spinal motor control (11). Another study reported that in patients with chronic low back pain, the somatotopic representations in S1 shift by 2.5 cm, and changes in gray matter volume are associated with pain chronicity. Additionally, non-painful afferents are suppressed, and cortical excitability decreases (19). In another study, individuals with chronic low back pain who received painful intradermal electrical stimulation showed that the S1 region shifted inward compared to healthy individuals (1). Some studies have also shown that in chronic pain, decreased activity in S1 and S2 is accompanied by increased activity in the prefrontal cortex, indicating why emotional, motivational, and cognitive states are more pronounced in chronic pain (19). Studies suggest that S1 and the posterior-external part of the prefrontal cortex can function as two neural networks in motor planning, movement execution, motor control, and pain management (21).

Regarding motor processing, studies indicate that individuals with chronic low back pain exhibit decreased excitability in M1 compared to healthy individuals (6). Other studies show that these individuals experience decreased M1 excitability and reduced GABA receptor activity (11), and those with decreased M1 cortical excitability experience significantly more pain immediately after pain onset and have slower recovery compared to those with higher M1 excitability (19).

Other studies also support that paraspinal muscle excitability, including multifidus, is reduced in individuals with chronic low back pain (22, 23) and the motor cortex map of trunk muscles undergoes reorganization. These studies reveal a connection between cortical changes and pain intensity and motor control (24). Additionally, it has been reported that in these individuals, the M1 area related to transverse abdominis muscle control shifts outward and posteriorly, which is associated with delayed activation of the transverse abdominis during rapid shoulder flexion (23). This delay in abdominal muscle reflexes may cause instability in spinal stabilization (25). Other studies have shown that cortical representations of multifidus and transverse abdominis muscles in healthy individuals are asymmetric, but in individuals with chronic low back pain, representations of the transverse abdominis muscle are more symmetrical and located predominantly in the left motor cortex (26). Paraspinal muscles receive descending inputs from the motor cortex and corticospinal pathways. These pathways not only cooperate in voluntary activity but also play a crucial role in postural control. Human studies have shown that postural activity of the trunk during limb movements decreases when the motor cortex is inhibited (24). Cortical changes cause the paraspinal fascicles to lose separate functional activity and act collectively. In other words, these muscles lose their ability to control independently and differentially, leading to similar activity between the longissimus and multifidus muscles. Consequently, postural deficits arise. Findings indicate that patients with more than 6 months of low back pain have a reduced M1 map in both acute and chronic phases. The reduced volume related to paraspinal muscles reflects decreased motor cortex excitability. Consequently, decreased motor cortex excitability results in limitations in reactive and anticipatory movements, aimed at minimizing pain and preventing further injury (27). This mechanism may reduce pain in the short term but can increase structural stress, reduce movements over time, and alter motor patterns, contributing to the persistence of low back pain (28). Overall, altered motor function and changes in the central nervous system due to low back pain may lead to impairments in spinal control and contribute to its persistence and recurrence (6, 29).

**2. General Objective**

The impact of combining sensory-motor exercises with transcranial direct current stimulation (tDCS) on brain sensory-motor processing and clinical symptoms in individuals with chronic low back pain.

**3. Descriptive Objectives**

**Primary:**

- To determine the average level of lumbar movement control in the groups (intervention and control), before and after the intervention.
- To determine the average level of disability in the groups (intervention and control), before and after the intervention.
- To determine the average level of pain in the groups (intervention and control), before and after the intervention.

**Secondary:**

- To determine the average amplitude of N80 and N150 in the groups (intervention and control), before and after the intervention.
- To determine the average active motor threshold of the multifidus and transverse abdominis muscles in the groups (intervention and control), before and after the intervention.
- To determine the average amplitude of the motor-evoked potentials (MEPs) of the multifidus and transverse abdominis muscles in the groups (intervention and control), before and after the intervention.

**4. Analytical Objectives**

**Primary:**

- To compare the average level of lumbar movement control in the groups (intervention and control), between before and after the intervention.
- To compare the average level of lumbar movement control between the groups (intervention and control) before and after the intervention.
- To compare the average level of disability in the groups (intervention and control), between before and after the intervention.
- To compare the average level of disability between the groups (intervention and control) before and after the intervention.
- To compare the average level of pain in the groups (intervention and control), between before and after the intervention.
- To compare the average level of pain between the groups (intervention and control) before and after the intervention.

**Secondary:**

- To compare the average amplitude of N80 and N150 in the groups (intervention and control), between before and after the intervention.
- To compare the average amplitude of N80 and N150 between the groups (intervention and control) before and after the intervention.
- To compare the average active motor threshold of the multifidus and transverse abdominis muscles in the groups (intervention and control), between before and after the intervention.
- To compare the average active motor threshold of the multifidus and transverse abdominis muscles between the groups (intervention and control) before and after the intervention.
- To compare the average amplitude of the motor-evoked potentials (MEPs) of the multifidus and transverse abdominis muscles in the groups (intervention and control), between before and after the intervention.
- To compare the average amplitude of the motor-evoked potentials (MEPs) of the multifidus and transverse abdominis muscles between the groups (intervention and control) before and after the intervention.
- To examine the correlation between clinical parameters and brain neurophysiological parameters.

**5. Research Questions and Hypotheses**

- Is there a difference in the average amplitude of N80 and N150 in the groups (intervention and control) between before and after the intervention?
- Is there a difference in the average amplitude of N80 and N150 between the groups (intervention and control) before and after the intervention?
- Is there a difference in the average active motor threshold of the multifidus and transverse abdominis muscles in the groups (intervention and control) between before and after the intervention?
- Is there a difference in the average active motor threshold of the multifidus and transverse abdominis muscles between the groups (intervention and control) before and after the intervention?
- Is there a difference in the average amplitude of the motor-evoked potentials (MEPs) of the multifidus and transverse abdominis muscles in the groups (intervention and control) between before and after the intervention?
- Is there a difference in the average amplitude of the motor-evoked potentials (MEPs) of the multifidus and transverse abdominis muscles between the groups (intervention and control) before and after the intervention?
- Is there a difference in the average level of lumbar movement control in the groups (intervention and control) between before and after the intervention?
- Is there a difference in the average level of lumbar movement control between the groups (intervention and control) before and after the intervention?
- Is there a difference in the average level of disability in the groups (intervention and control) between before and after the intervention?
- Is there a difference in the average level of disability between the groups (intervention and control) before and after the intervention?
- Is there a difference in the average level of pain in the groups (intervention and control) between before and after the intervention?
- Is there a difference in the average level of pain between the groups (intervention and control) before and after the intervention?
- Is there a significant correlation between clinical parameters and brain neurophysiological parameters?

**6. Practical Implications**

If combining exercises with tDCS yields better results, this method could be recommended for clinical use. If there is little difference between the treatment groups, it could be suggested that exercises alone are effective, thus avoiding additional costs, or seeking a new treatment method.

**7. Novelty of the Study**

Studies indicate that tDCS and sensory-motor exercises may positively affect pain reduction in patients with chronic low back pain. However, as noted, sensory and motor processing in the brain is disrupted in these patients. This aligns with other behavioral evidence indicating issues in sensory-motor control of the back in these individuals, which can ultimately lead to disability. Sensory-motor control is considered a key factor in the progression and recurrence of low back pain. Despite the significance of brain changes in sensory and motor areas and disruptions in sensory-motor control, no study has yet examined the effects of anodal tDCS on sensory and motor areas combined with sensory-motor exercises on brain processing and improvement in sensory-motor control in these patients. This study aims to assess for the first time the impact of anodal tDCS on the M1 and S1 regions, combined with exercises, on improving the mentioned parameters. The goal is to determine whether stimulating these regions with anodal tDCS, combined with exercises, can be a more effective treatment for individuals with chronic low back pain, considering the decreased excitability of the M1 and S1 areas in these individuals. The study will also focus on neurophysiological parameters in addition to clinical parameters, seeking to uncover potential mechanisms and understand the relationship between improvements in clinical parameters and changes in neurophysiological parameters, which has not been addressed before.

**8. Review of Previous Studies**

This section reviews previous articles in several subcategories:

- Effect of Combining Transcranial Electrical Stimulation (tDCS) with Sensory-Motor Exercises
- Effect of Combining tDCS with Other Exercises
- Effect of tDCS on Pain
- Effect of Sensory-Motor Exercises on Brain Reorganization
- Effect of Sensory-Motor Exercises on Pain and Muscular Function

**Effect of Combining tDCS with Sensory-Motor Exercises:**

- Adam Louis Ouellette and colleagues published a protocol in 2017 for a double-blind clinical trial. In this study, 80 patients with chronic low back pain were randomly assigned to two groups: real tDCS + sensory-motor exercises and sham tDCS + sensory-motor exercises. tDCS was applied for 20 minutes to the primary motor cortex along with 60 minutes of sensory-motor exercises twice a week for a total of 10 weeks. Feasibility (including the number of treatment sessions per patient, dropout rates, participant interest in continuing treatment, and completion of home exercise sessions), safety, pain, disability, and individual performance before and after the 10-week treatment were evaluated. The goal was to assess the efficacy of this treatment protocol (31).

**Effect of combining tDCS with Other Exercises:**

- Jafarzadeh and colleagues in 2019 studied the short-term and long-term effects of combining anodic tDCS and postural exercises in patients with low back pain and postural disorders. 38 individuals were divided into three groups: real anodic tDCS with exercises, sham anodic tDCS with exercises, and exercises alone. All participants received postural exercises three times a week for 20 minutes over two weeks. Anodal current was applied with 2 milliamperes for 20 minutes to the M1 area. Postural stability, balance, and pain were measured before, immediately after, and one month after the intervention. Significant improvements in balance, stability, and pain were observed in the group receiving real anodic tDCS with postural exercises, with no differences in other groups. The results indicate that anodic tDCS combined with postural exercise is effective in improving balance, stability, and pain in patients with low back pain and postural disorders, whereas postural exercises alone are insufficient to improve postural disorders (38).
- A double-blind pilot clinical trial by Sofia Straudi and colleagues in 2018 involved 35 patients with non-specific chronic low back pain receiving either five sessions of real or sham brain stimulation combined with ten sessions of group exercise. VAS, Rolland Morris questionnaire, EuroQuol-5, and patient health questionnaires were assessed. A significant difference in pain intensity and patient health questionnaires was found one month after combined treatment (39).
- A protocol study by Cavalcante and colleagues in 2020 examined the combination of tDCS with exercise therapy. They predicted that 60 patients with chronic low back pain would receive combined treatment (1-real tDCS + exercises or 2-sham tDCS + exercises) over 12 sessions across 4 weeks. Pain intensity, emotional and sensory aspects of pain, physical function, and fear of movement were assessed before, after, 3 months, and 6 months post-treatment. The exercises included stretching, strengthening, and motor control (40).
- A pilot double-blind study by Yuanbo Ma and colleagues in 2020 investigated the effect of high-definition tDCS and strengthening exercises for the plantar sensation and dynamic balance in individuals with chronic ankle instability. 30 adults were treated for four weeks with tDCS applied to facilitate excitability in the M1 and S1 areas. The combination of treatments improved dynamic balance and proprioception (41).
- A double-blind study by Mendonca and colleagues in 2016 aimed at modulating the motor system in fibromyalgia patients combined tDCS on M1 with aerobic exercises. 45 patients were divided into three groups (1-exercise + tDCS, 2-exercise only, 3-tDCS only). Dependent variables included pain intensity, anger, quality of life, mood, pressure pain threshold, and cortical plasticity. No significant differences in cortical plasticity were found among the groups, but the combination of tDCS and exercise had significant effects on pain, anger, and mood (42).

**Effect of tDCS on Pain:**

- In a double-blind study by Fuad Ahmad Hazime and colleagues in 2017, 92 patients with non-specific chronic low back pain were divided into four groups: (1-real tDCS + real peripheral stimulation; 2-real tDCS + sham peripheral stimulation; 3-sham tDCS + real peripheral stimulation; 4-sham tDCS + sham peripheral stimulation). Treatments were administered over 4 weeks with three sessions per week (total 12 sessions). Pain was assessed before, during, and after sessions. Real tDCS was applied at 2 milliamperes for 20 minutes, with active electrodes on C3 and C4, and sham electrodes on the supraorbital area. Peripheral stimulation was applied as a non-symmetric, rectangular biphasic current with 100 Hz frequency and 200 microseconds pulse duration for 40 minutes on the most painful part of the back. The study concluded that combining tDCS with peripheral stimulation could be effective in reducing low back pain in the long term, whereas tDCS alone did not significantly reduce pain (20).
- Timothy Y. Mariano and colleagues in 2018 conducted a double-blind study involving 30 patients with chronic low back pain divided into placebo and control groups. During ten treatment sessions, cathodal tDCS at 2 milliamperes for 20 minutes was applied to the FC1 area. Improvements in pain and depression were observed after treatment (43).

**Effect of Sensory-Motor Exercises on Brain Reorganization:**

- Bae and colleagues in 2014 studied 14 individuals (7 healthy and 7 with chronic low back pain). The study aimed to examine the effect of sensory-motor exercises on anticipatory postural control settings, brain changes in motor cortex areas using EEG, and the onset of contraction in the transverse abdominal and external oblique muscles using EMG, as well as pain. Patients with chronic low back pain were in the experimental group and performed exercises for 4 weeks, 4 sessions per week. Significant changes were observed in EEG parameters including readiness potential, motor potential, and movement monitoring potential. Reductions in readiness and motor potentials were noted in the relevant motor cortex areas, and significant differences in the onset of contraction in both muscles were found. Pain levels also decreased significantly (33).
- Rocco Cavaleri and colleagues in 2020 randomly divided 30 healthy individuals into two groups. Participants attended a single visuomotor exercise session divided into three phases (baseline, test, and recovery). Corticomotor responses were assessed twice in the baseline phase. In the test phase, one of two exercises (lumbo-pelvic tilt for the test group or repeated finger abduction for the control group) was performed for 15 minutes. Corticomotor responses were evaluated using TMS. In the recovery phase, the persistence of any corticomotor adaptation was assessed at 15 and 30 minutes post-exercise. The study found no significant correlation between corticomotor organization and performance improvements, suggesting that changes were more related to subcortical or spinal networks rather than corticomotor pathways. The researchers recommended further studies with varying complexities and durations (44).

**Effect of Sensory-Motor Exercises on Pain and Muscular Function:**

- Maryam Nazari Dehbazegi and colleagues in 2015 divided 53 patients with non-specific chronic low back pain into control and experimental groups. Sensory-motor exercises using a Huber device were performed for 5 weeks, with two 30-minute sessions per week. Significant improvements in lumbar movement control (Lomaguchi movement control tests) and pain reduction were observed in the sensory-motor exercise group compared to the control group (29).
- Michael A. McCaskey and colleagues in 2018 randomly assigned 22 patients with chronic low back pain to experimental and control groups. Both groups received routine physiotherapy for 30 minutes, but the experimental group also performed 15 minutes of sensory-motor postural exercises, while the control group performed 15 minutes of low-intensity cardiovascular exercises. Pain and function were assessed using the Oswestry Disability Index. Sensory-motor postural exercises had a significant impact on function but no significant effect on pain reduction or improvement in performance between the two groups with moderate pain (45).
- Jin Ah Hwang and colleagues in 2013 studied the effect of sensory-motor exercises on anticipatory postural control settings in chronic low back pain patients. Three groups were included: one group received conventional physiotherapy, another group received sensory-motor exercises, and the third group was a control group. The experimental groups performed their respective exercises for 40 minutes, 5 sessions per week for 4 weeks. Significant changes in pain, disability, and the onset of contraction in the transverse abdominal and external oblique muscles were observed. Sensory-motor exercises helped patients learn muscle control, resulting in reduced pain and improved function (46).
- Nisha Kanabar in 2016 examined pain and function changes using the Oswestry Disability Index. Patients were divided into two therapeutic groups: sensory-motor exercises (13 patients) and stability exercises (15 patients). Both groups performed exercises for 4 weeks, 5 sessions per week. Both types of exercises were effective in reducing pain and improving function in chronic non-specific low back pain patients, although sensory-motor exercises showed better effects (30).
- Benedict M. Wando and colleagues in 2011 conducted a preliminary study with three patients with chronic non-specific low back pain. The study aimed to evaluate the effectiveness of sensory-motor exercises combined with conventional physiotherapy. The results showed improved postural control, decreased pain, and increased lumbar flexion and extension, indicating the efficacy of sensory-motor exercises in combination with conventional physiotherapy (47).

This review highlights that the combination of tDCS and sensory-motor exercises shows promise in improving various outcomes, including pain, balance, and motor control. The effectiveness of these combined treatments varies across studies, and more research is needed to confirm their efficacy and establish optimal protocols for different patient populations.

**9. Study Type**

Randomized, single-blind clinical trial

**10. Study Population**

Target Population: Patients with chronic low back pain

Study Population: Patients with chronic low back pain who are accessible

**11. Sampling Method**

Samples will be selected through non-probability, voluntary sampling based on inclusion and exclusion criteria.

**12. Study Duration and Location**

From March 2022 to august 2023

Faculty of Rehabilitation, Tehran University of Medical Sciences – Biomechanics Laboratory and also the Brain Mapping Center of Iran

**13. Sample Size**

The sample size was calculated using G*Power 3.1.3 software, based on the Jafarzadeh et al. study [28], with pain as the primary outcome measure. The calculation used a between-group Cohen's f of 0.473, a power of 0.8, and an α of 0.05. This power analysis indicated that at least 30 participants were needed. To account for a potential 15% dropout rate, 34 participants were recruited.

**14. Inclusion Criteria**

- Age between 20 and 50 years
- Both male and female genders
- Chronic low back pain for more than 6 months, or 3 episodes of low back pain lasting more than one week in the past 12 months
- Unilateral radicular pain secondary to disc herniation at L4/L5 and L5/S1, confirmed by MRI
- Positive result in at least one of the tests: Slump test, Straight Leg Raise, or Lasegue’s sign
- Pain radiation from the anterior posterior calf to the area behind the leg associated with the L4/L5 dermatome to the posterior calf to the heel and outer part of the foot
- Average pain intensity must be 4 or more according to the Numerical Pain Scale
- Average disability score based on the Oswestry Disability Index must be 4 or more
- No spinal tumors
- No cognitive disorders according to Mini Mental Status Examination score ≥ 24
- No spondylolisthesis or spondylolysis
- No structural disorders or spinal deformities such as scoliosis, kyphosis, or severe lordosis
- No spinal fractures
- No brain injuries, including concussions or strokes
- No neurological diseases such as Parkinson’s disease, Alzheimer’s disease, or cerebellar disorders
- No scalp skin lesions or cuts
- No sensory disturbances or loss
- No history of epilepsy
- Not pregnant
- No metallic implants in the brain
- No skin infections
- No pacemakers or other implanted devices
- No visual disturbances
- No depression requiring medication
- No previous spinal surgeries

**15. Exclusion Criteria**

- Participant unwilling to complete or continue the study
- Absence from two consecutive or three non-consecutive treatment sessions
- Participation in a specific exercise program for back pain
- Use of narcotics, sedatives, or analgesics
- Skin irritation or sensitivity in the scalp area during sessions

**16. Data Collection Tools**

- Recording sensory evoked potentials with the EMG/NCV/EP5000 Q device
- Recording motor evoked potentials with the TMS MagPro X100 device
- Assessment of low back motor control using Luomajoki clinical tests
- Measurement of disability using the Persian version of the Oswestry Disability Index questionnaire
- Measurement of pain with the Visual Analog Scale (VAS)
- Cognitive assessment using the Mini Mental Status Exam (MMSE)
- Recording demographic and clinical characteristics with a designed questionnaire
- Consent form

**17. Procedure**Initially, the study will receive an ethics code and will be registered in the IRCT system to obtain a clinical trial code. Participants who meet the study criteria will be given detailed explanations about the research procedures so they have a full understanding of how the study will be conducted. Each participant will provide informed consent by signing a written consent form approved by the Tehran University of Medical Sciences Ethics Committee. Assessments will be conducted first, followed by treatments in the control group (sensory-motor exercises combined with sham transcranial electrical stimulation) and the intervention group (sensory-motor exercises combined with real transcranial electrical stimulation) for 4 weeks. Reassessment will occur 24 to 48 hours after the last treatment session.

**17-1. Assessment**
Assessments will be performed before the first treatment session and 24 to 48 hours after the completion of the treatment. Two laboratory tests and one clinical test will be conducted, and two questionnaires will be completed.

**17-1-1. Laboratory Assessments**
The laboratory assessments will include two tests: one to evaluate brain sensory processing and the other to evaluate brain motor processing.

**17-1-1-1. Test for Brain Sensory Processing**
To assess brain sensory processing, sensory evoked potentials will be recorded using the EMG/NCV/EP5000 Q device. Generally, sensory evoked potentials evaluate the sensory nervous system by applying somatosensory stimuli to record brain waves using electroencephalography electrodes. Electroencephalography reflects the brain's spontaneous electrical activity over a short period, whereas sensory evoked potentials are not recorded spontaneously but are associated with pre-stimulation.

In this test, two variables (amplitude N80 and N150) will be analyzed. Electrophysiological research shows that sensory evoked potentials recorded by electroencephalography reflect the processing of afferent sensory pathways in distinct areas of the brain cortex. The N80 time window is believed to represent processing in the primary sensory cortex (S1), while the N150 time window reflects processing in the secondary sensory cortex (S2).

For the test, participants will sit comfortably on a chair with their feet on the ground and hands relaxed. Participants will be asked to sit with their eyes closed but remain awake during the test. Sensory evoked potentials will be recorded using surface electrodes placed on the scalp. These electrodes will be positioned on the S1 area, 3 centimeters lateral to Cz and 2 centimeters posterior, and on the opposite side of the participant’s pain location. The scalp at the recording site should not have more than 5 kilo-ohms resistance. The reference electrode will be placed at Fz and the ground electrode on the forehead.

Bipolar electrodes for stimulation will be placed on the lower back, 3 centimeters lateral to the spinous process of L3, on the same side as the most pain. Electrical stimuli will have a pulse duration of 1 millisecond and will be applied at a frequency of 2 Hz. The bandwidth will be set from 1 to 500 Hz. Stimulation intensity will start at 1 milliampere and increase by 1 milliampere until the perceptual threshold is reached. Then, the stimulation intensity will be set to three times the perceptual threshold. If this intensity causes pain, the stimulation intensity will be decreased by 1 milliampere until it is no longer painful. Stimuli will be applied 500 times, and this process will be repeated twice, with the average of the two repetitions used in the final analysis. The maximum amplitude of sensory evoked potentials will be below 10 microvolts. The amplitude N80 is the largest peak in the 40 to 90 milliseconds range from the onset of the wave, while the amplitude N150 is the largest peak in the 90 to 180 milliseconds range, though in this study, N80 and N150 were observed more in the 65 and 120 milliseconds ranges. As shown in the figure, the maximum amplitude is the difference between the highest peak and the lowest trough.


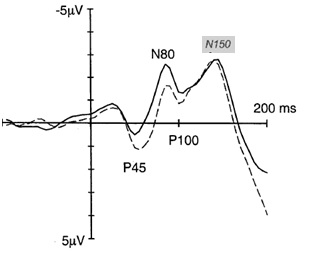
Top of Form

Bottom of Form

Fig 1. Sensory-evoked potentials (N80 and N150)


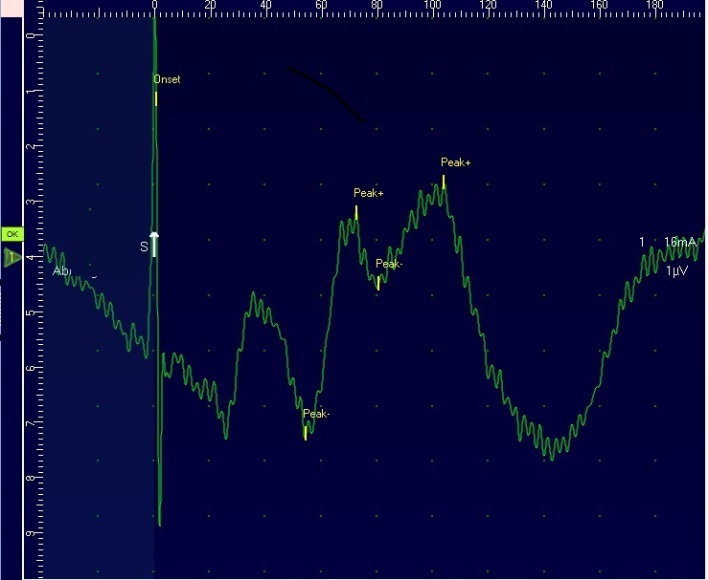


Fig 2. N80 and N150 Amplitudes

**17-1-1-2. Test for Brain Motor Processing**

To assess brain motor processing, motor threshold and motor evoked potentials will be measured using the TMS MagPro X100 device. Generally, the goal of motor evoked potentials is to evaluate the motor nervous system by applying a single stimulating pulse through transcranial magnetic stimulation to the motor cortex and then recording this motor stimulation from the corresponding muscle using electromyography electrodes. The recorded wave is known as the motor evoked potential.

Motor threshold will also be an index of cortical excitability and will be used to determine the intensity of stimulation with the device.

In this test, four variables (active motor threshold and amplitude of motor evoked potentials for the multifidus muscle and abdominal transverse) will be examined. These variables are indicators of excitability, performance, and the integrity of neural pathways and corticospinal areas related to these muscles.

For recording variables related to the multifidus muscle, the patient will sit comfortably on a chair with their feet on the ground. A surface electromyography electrode will be placed on the spinous process of L5, aligned with a line connecting the PSIS and the L1-L2 space. The ground electrode will be placed on the iliac crest. The bandwidth will be set from 5 to 500 Hz. To achieve maximal contraction in the multifidus muscle, the participant will be asked to perform a trunk extension against resistance and hold it for 3 seconds. This movement will be repeated 3 times, and the maximum contraction will be measured based on the Root Mean Square (RMS) within a 1-second window. Then, 20% of this value will be used as the submaximal contraction, which will be displayed on a monitor for the participant to maintain throughout the recording. This contraction is intended to facilitate the recording of motor evoked potentials in the paravertebral muscles. In other words, if the muscle is in a resting state, recording will be difficult.

To record variables related to the abdominal muscles, the patient will sit comfortably on a chair with their arms resting on the chair and knees apart. The surface electromyography electrode will be placed 2 cm below and 2 cm inward from the ASIS, which are the most superficial parts of these muscles, and the ground electrode will be placed on the iliac crest. The bandwidth will be set from 20 to 450 Hz. To achieve maximal contraction in the abdominal muscles, the participant will be asked to perform a forced exhalation maneuver, which will be held for 3 seconds and repeated 3 times. Then, 15% of this contraction will be considered as submaximal and should be maintained throughout the recording.

For stimulation, a double-cone coil will be used. This coil is ideal for stimulating the motor parts of the trunk muscles. It will be placed in the M1 region, as evidence suggests that this point is the best for stimulating cortical pathways and motor nerves, resulting in better-evoked potentials. Based on previous studies, the motor cortex related to trunk muscles is recommended to be 2 cm lateral from the midline and 2 cm anterior to the vertex. Stimulation will be applied on the side opposite to the area of the back with the most pain. The coil will be positioned at a 45-degree angle to the anteroposterior axis to apply a current from back to front.

Initially, the active motor threshold will be determined. The active motor threshold is the lowest TMS intensity that generates a motor potential with a minimum amplitude of 100 µV in at least 5 out of 10 trials. Since recording occurs during isometric muscle contraction, it is termed "active." Typically, the initial stimulation intensity is set at 25% of the maximum output of the device and then gradually increased by 2% until the desired recording characteristics are achieved. The distance between the scalp and the coil will be less than 5 mm.

After determining the active motor threshold, the coil intensity will be set to 120% of the active motor threshold, and the amplitude of the resulting evoked potential will be measured. The amplitude of the active motor evoked potential will be defined as the difference between the highest peak and the lowest trough.


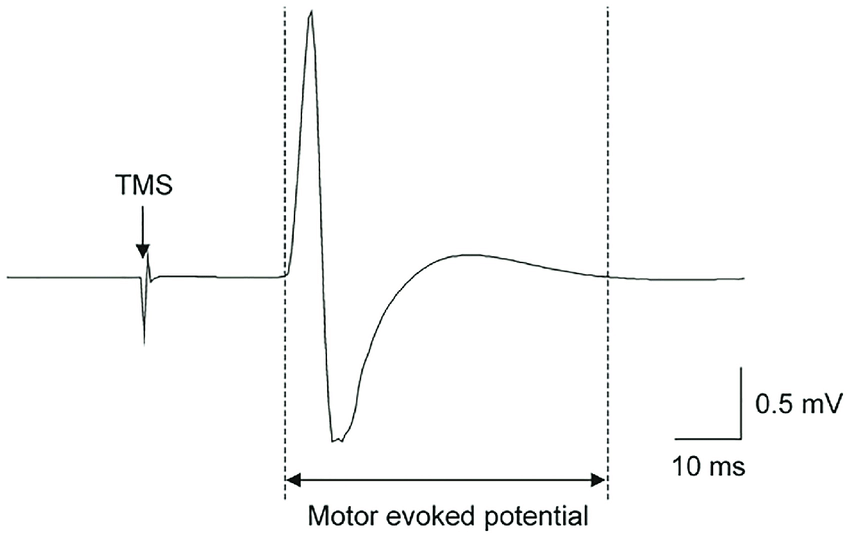


Fig 3. Motor-evoked potentials

**17-1-2. Clinical Assessments**

**17-1-2-1. Trunk Motor Control**

To assess trunk motor control, six tests designed by Luomajoki, which have a high validity (0.6), will be used. These tests are reliable tools for diagnosing disorders in lumbopelvic control. First, the examiner explains how to perform the movement and asks the individual to do it. If the movement is not performed correctly or the person does not understand how to perform it, the examiner will attempt to correct the movement with additional explanations. If the movement is still not performed correctly in the second attempt, the examiner will demonstrate the correct movement. If the individual cannot perform the movement correctly after the second attempt, they will receive a score of 1, and if performed correctly, a score of 0. A score of 0 indicates that all tests were performed correctly, while a score of 6 means none of the tests were performed correctly. Generally, each movement is allowed to be performed three times.

**17-1-2-2. Disability Level**

To assess disability, the Persian version of the Oswestry Disability Index questionnaire will be used. This questionnaire is designed to evaluate functional disability in individuals with low back pain and includes ten sections, such as pain intensity, personal care, walking, sitting, and others. In each section, individuals receive a score from 0 to 5. The total score is calculated numerically. A score of 0-4 indicates no disability, 5-14 indicates mild disability, 15-24 indicates moderate disability, 25-34 indicates severe disability, and 35-50 indicates complete disability (30). The questionnaire is available in Appendix 3.

**17-1-2-3. Pain Level**

To evaluate pain intensity, the Visual Analog Scale (VAS) will be used. This scale consists of a 10 cm line, where the individual rates their pain from 1 to 10. Zero represents no pain, and ten represents unbearable pain (48). This scale is a discrete quantitative measure used to assess changes in pain intensity before and after interventions.

**17-2. Treatments**

After the initial assessment, individuals will be randomly assigned to one of two groups. The sequence will be generated using the website randomization.com with a balanced block randomization method. The block size will be 4. To ensure allocation concealment, sealed and numbered envelopes will be used, which will be provided to participants by the secretary. The treatment period for both groups will be 12 sessions, three times a week. After the treatment period, evaluations will be conducted again.

Due to the inclusion of a sham treatment, participants will be blinded, but blinding of the therapist or evaluator will not be possible.

**17-2-1. Control Group**

In this group, participants will receive sensory-motor exercises combined with sham transcranial electrical stimulation for 4 weeks, three times a week (12 sessions). Participants will first receive the sham stimulation and then perform the exercises.

**17-2-2. Intervention Group**

In this group, participants will receive sensory-motor exercises combined with real anodal transcranial electrical stimulation for 4 weeks, three times a week (12 sessions). Participants will first receive the real stimulation and then perform the exercises.

**17-2-3. Sensory-Motor Exercises**

The goal of sensory-motor exercises is to enhance proprioceptive input from three areas (the soles of the feet, the sacroiliac joint, and the cervical vertebrae) to facilitate coordinated and automatic movement patterns. Therefore, maintaining the correct positioning of these three areas is essential throughout the exercises. To stimulate the foot receptors, exercises will be performed barefoot. Participants will then be asked to contract the muscles of the feet to increase the arch of the foot without bending the toes. Initially, for those unable to contract the foot muscles, a tape strip will be applied to assist in maintaining the foot in the correct position.

During the exercises, the sacroiliac joints and cervical vertebrae must also be in a neutral position. Participants will be asked to slightly pull in their navel to facilitate the function of the transverse abdominal muscles. Additionally, the chin tuck maneuver will activate the deep neck flexor muscles. The exercises will generally be performed in two phases (static and dynamic).

**Static Phase**

In this phase, the focus will be on pelvic stability through the contraction of the diaphragm, multifidus, pelvic floor, and transverse abdominal muscles to provide a foundation for performing limb movements in subsequent stages. This phase is based on the principle of "proximal stability for distal movements." Progression in this phase will be from standing on two feet, standing on one foot, and then standing in a half-step position.

The support surface will initially be firm and then unstable. Foam, balance pads, rocker boards, and wobble boards will be used. The center of gravity will be challenged by disturbances or weight shifts using elastic bands, and the participant must maintain stability. These positions will elicit automatic postural and reflexive responses.

**Dynamic Phase**

Once the participant can maintain pelvic stability in the previous phase, they will advance to the dynamic phase, where they will perform upper and lower limb movements while maintaining pelvic stability. Progression in this phase will be similar to the static phase: standing on two feet, standing on one foot, and then standing in a half-step position. The support surface will be firm initially and then unstable. In the next stage, the center of gravity will be challenged using elastic bands and ball throws. These exercises will facilitate the retraining of feedforward mechanisms.

Overall, the progression of exercises will be based on each individual's ability, and each stage will be continued until the therapist determines that the patient has achieved the necessary stability and is ready for the next stage of exercises. In these exercises, the focus will be on the quality of performance rather than the quantity of exercises.

**17-2-4. Transcranial Electrical Stimulation**

To apply electrical current, the tDCS device model Neurostim2 from Medina Teb Gostar will be used. Before the patient's arrival, all equipment including electrodes, normal saline, stimulator, cables, elastic bands, and measurement tape will be inspected to ensure they are in good condition and free of defects or damage. The patient will sit on a chair, and the scalp will be examined for any lesions. The researcher will ask the participant to report any skin irritation from the previous session or anything else that would exclude them from the study.

Initially, the electrodes will be placed in sponges soaked with normal saline, and the area of stimulation will be cleaned with alcohol. The electrode size will be 2x4 cm. Stimulation will be applied through two active (anodal) electrodes placed on the scalp. One active electrode will be placed on the M1 region, corresponding to C3 or C4 according to the 10-20 international system. The other active electrode will be placed on the S1 region, which is 2 cm posterior to C3 or C4. Reference (cathodal) electrodes will be placed on the forehead directly above the eyebrow (Figure 6). In general, active electrodes will be placed on the opposite side of the lower back pain, and the reference electrode will be placed on the supraorbital area on the same side as the pain. The device will be set to a current intensity of 1.5 mA for 20 minutes. At the start of stimulation, there will be a 10-second ramp-up period during which the current will gradually increase to 1.5 mA. At the end, there will be a 10-second ramp-down period during which the current will gradually decrease and the device will turn off. Overall, considering the size of the active electrodes, the average current density under these electrodes will be 0.188 mA per square centimeter. The participant will be informed that they might experience a tingling or itching sensation, and the patient will be continuously monitored throughout the treatment. In the sham group, the electrodes will be placed similarly to the intervention group. The device will be turned on, and current will be applied for only 10 seconds, during which the participant will feel a tingling sensation, and then the current will be turned off. The duration of the current application in the sham group will also be 20 minutes (32).


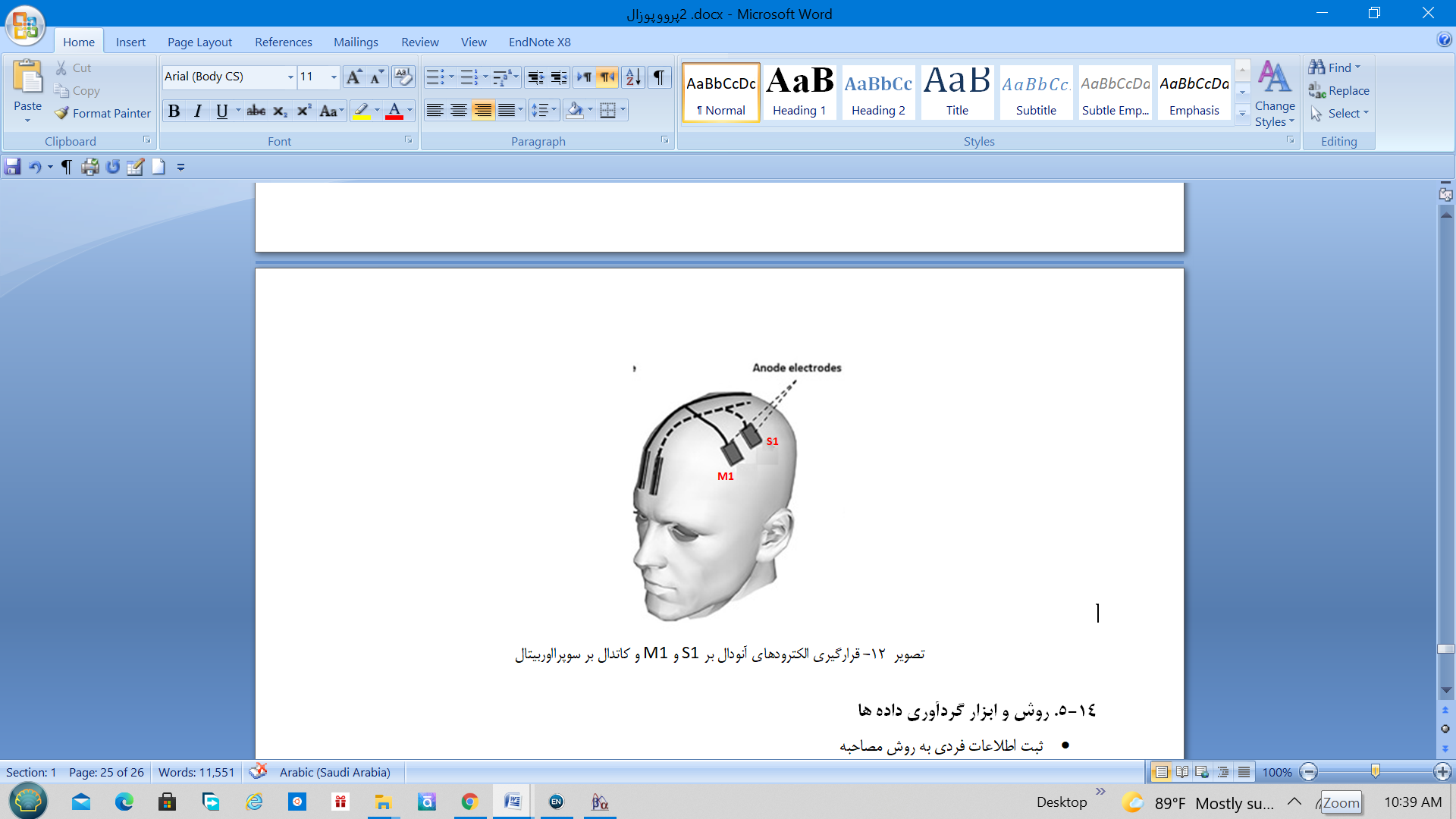


Fig 4. Placement of anodal electrodes on S1 and M1 and cathodal electrodes on the supraorbital area

**18. Data Analysis Method**

- All descriptive and statistical analyses will be conducted using SPSS software version 23.
- Descriptive statistics will include the calculation of measures of central tendency (mean) and dispersion (standard deviation).
- The Shapiro-Wilk test will be used to assess the normal distribution of the data.
- To compare baseline (pre-treatment) between the two treatment groups, an Independent t-test with a significance level of 0.05 will be used.
- To examine the effects of within-subject factors (time stages: pre- and post-treatment) and between-subject factors (type of treatment: real transcranial electrical stimulation with sensory-motor exercises vs. sham transcranial electrical stimulation with sensory-motor exercises), a Two-Way Mixed between-within ANOVA will be employed. Effect sizes will be reported using η², and interpreted as small (0.01), medium (0.06), and large (0.14).
- Pearson Correlation will be used to assess the relationship between clinical parameters and neurophysiological parameters related to sensory and motor cortical excitability.

**19. Ethical Considerations**

This study will be approved by the Ethics Committee of Tehran University of Medical Sciences and will receive an ethics code. Before conducting the test, all procedures, methods, and objectives will be clearly explained to the participants. Each participant will provide informed consent by completing a consent form and may withdraw from the study at any stage if they choose not to participate. Participants will be assured that the evaluation process will incur no costs, and appropriate measures will be taken to prevent any harm during the assessments. The information obtained from participants will be used solely for statistical analysis, and confidentiality will be maintained. The overall results of the study will be reported in aggregate form without identifying individual participants. Additionally, the tools used in this research are non-invasive and will not cause any harm to the participants. A recent study on the safety of tDCS indicated that among 1,000 individuals receiving 33,000 sessions of tDCS, no evidence of harm was found when tested with these parameters (≤ 40 min, ≤ 4 mA, ≤ 7.2 C).
