# Supplementary material for: Effects of combining sensory-motor exercises with transcranial direct current stimulation on cortical processing and clinical symptoms in patients with lumbosacral radiculopathy: An exploratory randomized controlled trial
Source: PLoS One. 2024 Dec 19;19(12):e0314361. doi: 10.1371/journal.pone.0314361 (PMC11658635; doi:10.1371/journal.pone.0314361)
Supplement: S2 File — (DOCX) [file pone.0314361.s003.docx]

**اثر ترکیب تمرینات حسی-حرکتی با تحریک الکتریکی فراجمجمه ای بر پردازش حسی- حرکتی مغز و علائم بالینی در افراد مبتلا به کمردرد مزمن**

**سهیلا قنبری، رویا خانمحمدی، غلامرضا علیایی، زهره حسینی، حانیه سادات حجازی**

## 1. مقدمه و بیان مسئله

در حال حاضر کمردرد یکی از رایج ترین مشکلات در سراسر دنیاست که با میزان شیوع 80 تا 85%، سبب ناتوانی فرد در طول زندگی میشود (1, 2). از بین این افراد 5 تا 10% به سمت کمردرد مزمن پیش می روند که سبب تحمیل هزینه های درمانی بالا و تحمل درد و رنج برای آنها می شود (3). کمردرد سبب محدود شدن تقریبا 39% از فعالیت ها در تمام دنیا شده است (4).

به طور کلی، کمردرد مزمن، کمردردی است که بیش از 3 تا 6 ماه به طول انجامد (5). درد مزمن یک سندروم پیچیده است که شامل اجزای عاطفی^[[1]](#footnote-2)^، روانی و مکانیکال است (6). علیرغم پیشرفت هایی که در ابزارها و تکنیک های تشخیصی وجود دارد، در بسیاری از موارد، دانستن علت دقیق علایم، غیر ممکن است. اما آنچه مشهود است، آن است که در شرایط مزمن، درمان تنها بر اساس اصلاح ناهنجاری های ساختاری در سیستم عضلانی- اسکلتی غالباً ناموفق است که نشان دهنده وجود دیگر مکانیسم های پاتوفیزیولوژیکال و بایوسایکوسوشال در کمردرد مزمن می باشد (1, 7).

بیش از 20 سال است که تحقیقات فزاینده ای در حال انجام هست که نشان میدهد افراد مبتلا به کمردرد، ممکن است تغییراتی در سیستم عصبی مرکزی خود داشته باشند (8). بسیاری از شواهد نشان می دهند که تغییرات ساختاری در سیستم عضلانی اسکلتی ناحیه کمر، ممکن است به تنهایی پاسخگو نباشد و در حقیقت یک سازماندهی مجدد^[[2]](#footnote-3)^ وسیع در کورتکس می تواند اتفاق افتاده باشد؛ مثل اختلالات نوروشیمیایی، عملکردی و ساختاری در مغز؛ که این تغییرات ممکن است در تداوم کمردرد نقش داشته باشند که بایستی از منظر درمانی نیزمورد توجه قرار بگیرند. به عبارتی، توجه به مکانیسم های پاتوفیزیولوژیکال کمردرد مزمن در شناسایی موثرترین روش درمان برای این دسته از افراد، کمک کننده می باشد (7). مطالعات اخیر نشان می دهد که تغییرات ساختاری و عملکردی در سیستم عصبی مرکزی (9) افراد مبتلا به دردهای عضلانی - اسکلتی، منعکس کننده فرآیندهای نوروفیزیولوژیکال انطباقی^[[3]](#footnote-4)^ است و از طریق محافظت از ساختارها به پروسه ترمیم کمک خواهد کرد. بر طبق یافته های آزمایشگاهی و کلینیکی، این تطابقات نوروفیزیولوژیکال ممکن است ماندگار شده و پیشرفت کنند و علایم مزمن از خود برجای بگذارند (10).

در حقیقت این پلاستیسیتی ناسازگار در سیستم عصبی مرکزی در پاسخ به درد، یکی از مهم ترین پدیده هایی است که نشان میدهد چرا افراد مبتلا به کمردرد مزمن به درمانهای سنتی به سختی پاسخ میدهند (11). نوروپلاستیسیتی یک فرایند نوروفیزیولوژیکال اساسی و ذاتی است که شامل یکسری تغییرات ساختاری،عملکردی و سازماندهی در مغز و سیستم عصبی است که به طور مداوم در تمام طول زندگی فرد اتفاق می افتد (10). مکانیسم اصلی نوروپلاستیسیتی در سیستم عصبی مرکزی، تغییرات عملکردی یا تداخلی در خواص ذاتی نورون ها و همچنین تغییرات ساختاری در تعداد یا محل سیناپس های بین نورونی میباشد (12). تطابق نوروپلاستیک، ابتدا از آسیب ساختاری عضلانی- اسکلتی نشات می گیرد و باعث می شود در شرایط مزمن، بدون حتی هیچگونه آسیب آناتومیکی/ساختاریِ عضلانی- اسکلتی، فرد شرایط پاتوفیزیولوژیکال را تجربه کند (10). در حقیقت پلاستیسیتی سیناپسی شامل بیان تغییرات ماندگار در کیفیت سیناپسی است که در نتیجه ی تقویت سیناپسی و یا تضعیف سیناپسی ایجاد می شود (13).

در این راستا شواهد نشان می دهد درد در ارتباط با پلاستیسیتی ناسازگار در کورتکس حسی و حرکتی سیستم عصبی مرکزی می باشد (14, 15). این یافته ها مکمل دیگر شواهد رفتاری است که اذعان دارند در عملکرد حسی- حرکتی ناحیه کمر بیماران، اشکالاتی وجود دارد که در نهایت می تواند باعث ناتوانی این افراد شود (1). آن چنان که کنترل حسی- حرکتی کمر به عنوان یکی از فاکتورهای کلیدی ممکن، در پیشرفت و رجوع کمردرد شناخته می شود (16).

رفتارهای حسی- حرکتی بهینه، وابسته به پردازش مرکزی دقیق و یکپارچه کردن اطلاعات حسی به فرامین حرکتی می باشد (16). در حقیقت رابطه پیچیده ای بین درک و عمل وجود دارد (1) و خروجی های حرکتی عموما وابسته به ورودی های حسی هستند. این ورودی های حسی شامل اطلاعات سوماتوسنسوری از وضعیت و حرکت بدن و اطلاعاتی از شبکه حس عمقی می باشد (8). اطلاعات حسی برای انتخاب استراتژی مناسب حرکتی، به منظور ایجاد ثبات و حرکت ستون فقرات، نقش مهمی دارند (1). به منظور حفظ پاسچر ستون فقرات و کنترل حرکت، تعامل مداوم بین برون ده حرکتی راه های عصبی مربوط به عضلات پارااسپاینال و ورودی های حسی از جمله حس عمقی در سطوح مختلف سیستم عصبی حائز اهمیت است (9, 17).

به طور کلی، درد، عملکرد حسی - حرکتی را تحت تاثیر قرار می دهد. زمانی که درد ظاهر می شود، ظرفیت تولید نیرو توسط عضلات کاهش می یابد؛ هماهنگی عضلانی دچار اختلال می شود (18)؛ الگوی بدن در حین کار تغییر می کند (7)؛ حس عمقی دچار اختلال می شود (1, 16)؛ دقت لمسی کاهش می یابد (1, 7, 11, 16) و همچنین توانایی یکپارچه سازی اطلاعات حسی به فرامین حرکتی کاهش می یابد (18). به عبارت دیگر اختلال در کنترل حسی- حرکتی که شامل تمامی پردازش های حسی و حرکتی است، که منجر به کنترل عضلات و حرکات و همچنین حفظ راستای ستون فقرات و در نهایت سبب عملکرد سالم و بارگذاری مناسب روی ستون فقرات می شود، به عنوان یک مکانیسمِ احتمالیِ زمینه سازِ توسعه یا پایداری درد، محسوب می شود (8).

همانطور که پیشتر اشاره شد، یکی از مکانیسم های پاتوفیزیولوژیکال مطرح شده برای اختلال در کنترل حسی- حرکتی کمر در افراد مبتلا به کمردرد، تغییر در پردازش حسی- حرکتی سیستم عصبی مرکزی می باشد. در حقیقت یافته ها نشان می دهد که در افراد مبتلا به کمردرد مزمن، سازماندهی مجددِ قسمتهایی از مغز که مربوط به پردازش اطلاعات حسی هستند، ممکن است در دقت حسی که در ارتباط با درک بدن^[[4]](#footnote-5)^ می باشد و در کنترل عصبی ناحیه کمری نقش مهمی ایفا می کند، دخیل باشد (1). به طور کلی سازماندهی مجدد سیستم حسی- حرکتی مغز ممکن است سبب ایجاد تغییراتی در تظاهرات حسی و حرکتی از جمله اختلال در تصور بدن^[[5]](#footnote-6)^ (10) و تغییر عملکردی در برنامه ریزی حرکتی و در نهایت ضعف کنترل پاسچرال شود (1).

در رابطه با پردازش حسی، نتایج مطالعات حاکی از آن است که در افراد مبتلا به کمردرد (هم در فاز حاد و هم در فاز مزمن) تغییر و اختلال در کورتکس حسی اولیه (S1) اتفاق می افتد و این تغییرات در ارتباط بسیار با شدت درد، نقص عملکرد و مزمن شدن علایم است (11, 19). در حین شرایط حاد یا شرایط درد عضلانی آزمایشی، کاهش تحریک پذیری^[[6]](#footnote-7)^S1 قبل از تغییر در برون ده حرکتی اتفاق می افتد(19). همچنین در بیمارانی که ناحیه S1 دچار کاهش تحریک پذیری می شود، نسبت به افرادی که این تحریک پذیری زیاد می شود، درد بیشتری احساس میشود (19). در درد مزمن نیز، پیشرفت خاطره درد می تواند نقشه سوماتوتوپیک S1 را تغییر دهد و باعث ایجاد حساسیت بالا حتی بدون محرک درد شود (12).

علاوه بر این، یک ارتباط معناداری بین چگالی ماده خاکستری مغز و شدت و مدت زمان درد وجود دارد (12). شواهد اخیر نشان داده است که کمردرد مزمن در ارتباط با کاهشماده خاکستری در کورتکس سوماتوسنسوری است (20). در این راستا، دیگر مطالعات نشان داده اند که در کمردرد مزمن، ضخامت کورتکس حسی اولیه (S1) و حسی ثانویه ((S2 در افراد مبتلا به کمردرد مزمن نسبت به گروه کنترل کاهش پیدا کرده است (11). ناحیه S1 سیگنال های حسی تک مدلی را پردازش کرده و با سیگنال های حرکتی ترکیب و یکپارچه می کند تا بتواند حرکت را هدایت کند. ناحیه S2 ورودی های حسی چند مدلی و دوطرفه را یکپارچه می کند و یک نقطه کلیدی در یکپارچه سازی حسی-حرکتی^[[7]](#footnote-8)^ در خلال ارتباطش با نواحی حرکتی محسوب می شود (16).S1 سبب دقیق شدن کنترل حرکتی نیزمی شود (17). همچنین مطالعات نشان داده اند که در افراد مبتلا به کمردرد، ناحیه S1که اطلاعات را از تنه دریافت می کند، در مغز دچار تغییر محل می شود؛ از این رو ارتباط با M1 مختل شده و کنترل حرکتی ستون فقرات کاهش می یابد (11). در مطالعه ای دیگر گزارش شده است که در بیماران مبتلا به کمردرد مزمن، تظاهرات سوماتوتوپیک در S1، 5/2 سانتی متر جا به جا شده است و تغییرات حجم ماده خاکستری در ارتباط با مزمن شدن درد می باشد. همچنین پردازش آوران های غیر درد، سرکوب شده و تحریک پذیری کورتکس حرکتی کاهش می یابد (19). در مطالعه ای دیگر، در افراد مبتلا به کمردرد مزمن که مغز توسط یک تحریک الکتریکی داخل جلدی دردناک تحریک شد، مشاهده شد که ناحیه S1 نسبت به افراد سالم به سمت داخل تغییر محل داده است (1). یکسری مطالعات هم نشان داده اند در درد مزمن، کاهش فعالیت S1، کاهش فعالیت S2 به همراه افزایش فعالیت کورتکس پری فرونتال وجود دارد و این خود بیانگر آن است که چرا در درد مزمن، حالت های عاطفی، انگیزشی^[[8]](#footnote-9)^ و شناختی^[[9]](#footnote-10)^ بصورت قوی تر ظاهر می شوند (19). در مطالعات نشان داده شده است که S1 و قسمت خلفی-خارجی کورتکس پری فرونتال^[[10]](#footnote-11)^، می توانند با یک دیگر، بصورت دو شبکه عصبی در برنامه ریزی حرکتی، اجرای حرکت، کنترل حرکت و همچنین مدیریت درد نقش مهمی ایفا کنند (21).

در خصوص پردازش حرکتی، نتایج مطالعات بیانگر آن است که در افراد مبتلا به کمردرد مزمن، کاهش تحریک پذیری در M1 نسبت به افراد سالم وجود دارد(6). مطالعات دیگر نیز نشان می دهد که این افراد به تحریک پذیری پایین M1 و کاهش فعالیت گیرنده گابا^[[11]](#footnote-12)^دچارند (11) و افرادی که با کاهش تحریک پذیری کورتکس M1 مواجه هستند، بلافاصله پس از شروع درد، درد بسیار بیشتری را تجربه می کنند و علاوه بر آن ریکاوری آهسته تری را نسبت به افرادی که تحریک پذیری بالای کورتکس M1دارند، خواهند داشت (19).

نتایج سایر مطالعات نیز موئد آن است که تحریک پذیری عضلات پارااسپینال ازجمله مالتی فیدوس در افراد مبتلا به کمردرد مزمن کاهش یافته (22, 23) و نقشه کورتکس حرکتی عضلات تنه تحت سازماندهی مجدد قرار گرفته است. در نتیجه این مطالعات مشخص شد که یک ارتباط بین تغییرات کورتیکال و تغییرات شدت درد و کنترل حرکتی وجود دارد (24). علاوه براین گزارش شده است که در این افراد ناحیه M1 مربوط به کنترل عضله عرضی شکمی به سمت خارج و خلف شیفت پیدا می کند که این شیفت، در ارتباط با تأخیر در فعالیت عرضی شکمی در فعالیت فلکشن سریع شانه است(23). این تأخیر در رفلکس های عضلات شکمی ممکن است سبب اختلال در ثبات ستون فقرات شود (25). سایر مطالعات نیز نشان داده اند تظاهراتکورتیکال عضلات مالتی فیدوس و عرضی شکمی در افراد سالم به صورت غیر قرینه است ولی در افراد مبتلا به کمردرد مزمن، تظاهراتمربوط به عضله عرضی شکمی، بیشتر در کورتکس حرکتی سمت چپ و به صورت قرینه است (26). عضلات پارااسپاینال، ورودی های پایین رونده را از کورتکس حرکتی و راه های کورتیکواسپاینال دریافت می کنند. این راه ها نه تنها در فعالیت ارادی همکاری می کنند بلکه حتی در کنترل پاسچرال هم نقش مهمی ایفا می کنند. در مطالعات انسانی نشان داده شده است که فعالیت پاسچرال تنه هنگام حرکات اندام ها، زمانی که کورتکس حرکتی مهار می شود، کاهش می یابد(24).تغییرات کورتیکال باعث می شود که فسیکل های پاراسپاینال، فعالیت عملکردی مجزا از هم را، از دست بدهند و بصورت دسته جمعی وارد عمل شوند. به عبارتی این عضلات توانایی کنترل مستقل و افتراقی^[[12]](#footnote-13)^خود را از دست میدهند؛ این امر سبب می شود که عضلات لانجیسیموس و مالتی فیدوس فعالیت مشابه هم را داشته باشند. در نتیجه، نقص پاسچرال ایجاد خواهد شد. یافته ها نشان می دهند مراجعه کنندگانی با بیش از 6 ماه کمردرد، نقشه M1 آنها در فاز حاد و مزمن، حجم کمی دارد. کوچک بودن حجم مربوط به عضلات پارااسپاینال، نمایانگر پایین بودن تحریک پذیری کورتکس حرکتی است. در نتیجه کاهش تحریک پذیری کورتکس حرکتی، محدودیت در حرکات واکنشی^[[13]](#footnote-14)^ و پیش بینانه ^[[14]](#footnote-15)^ خواهیم داشت. هدف از این حرکات به حداقل رساندن درد و جلوگیری از آسیب بیشتر است (27). این مکانیسم در کوتاه مدت می تواند از شدت درد بکاهد ولی در طولانی مدت سبب افزایش فشار روی ساختارها، کاهش حرکات به صورت طولانی مدت و تغییرات الگوهای حرکتی می شود (19). به طور خلاصه در کمردرد مزمن، کنترل حرکتی ناکارآمد می تواند در ارتباط با سازماندهی مجدد M1 و فیزیولوژی تغییر یافته کورتکس حرکتی باشد.

از منظر درمانی برای بهبود کنترل حسی-حرکتی کمر در افراد مبتلا به کمردرد مزمن، تمرین درمانی جزء خط مقدم درمان می باشد (28). در تحقیقات اخیر، تمرکز محققان بر روی تمرینات حسی- حرکتی^[[15]](#footnote-16)^ است که حس و حرکت را به طور هم زمان مورد هدف قرار می دهد (7, 29). این تمرینات سبب تحریک ارگان های حاوی حس عمقی و افزایش هماهنگی بین گروه های مختلف عضلانی و بهبود پاسخ نسبت به محرک های حسی می شود (28, 30). در حقیقت مکانیسم اثر این گونه تمرینات، بهبود درد از طریق نرمال کردن تغییرات حسی و حرکتی است (31). تمرینات حسی- حرکتی سبب افزایش ورودی های حس عمقی شده و در نتیجه پاسخ های حرکتی در محیط های داینامیک را بهبود می بخشد که در نهایت ممکن است سبب بهبود کیفیت کنترل پاسچرال و تسکین درد های مختص عضلانی- اسکلتی شود (28, 32). تمرینات حسی- حرکتی می تواند سبب بهبود کنترل حرکتی در محیط های پیچیده و چالش برانگیزی شود که دارای محرک های غیر قابل انتظار هستند (28). مطالعه Sea-Hyun Bae در سال 2014 نشان داد که در افراد مبتلا به کمردرد مزمن، تمرینات حسی- حرکتی می تواند فعالیت مغزی ثبت شده بوسیله الکتروانسفالوگرافی را تحت تاثیر قرار دهد. در حقیقت این نوع تمرینات می توانند توانایی کنترل عضلانی را به واسطه نوروپلاستیستی در کورتکس مغزی بهبود بخشند. این تمرینات درافراد سالم و مبتلا به کمردرد با تنظیم ازپایین به بالا، باعث کاهش درد می شود (33). اما همان طور که قبلا اشاره شد در کمردرد مزمن، بسیاری از مراکز مغزی شامل مناطق حسی- حرکتی کورتکس، دچار کاهش تحریک پذیری شده اند. از این رو نیازمند استفاده از درمان هایی هستیم که بتواند روی تحریک پذیری کورتکس تغییرات پایدار ایجاد کند تا بیشترین تاثیر را روی کمردرد مزمن نسبت به روش های درمانی سنتی داشته باشیم (34) و در این راستا افزودن درمان هایی که با تنظیم از بالا به پایین باعث بهبود عملکرد می شوند، حائز اهمیت است. همچنین از سوی دیگر، دیدگاه های نوین بر آن است که درمان های ترکیبی و چند فاکتوره نسبت به درمان های تک بعدی دارای اثر بخشی بیشتری هستند. تحریک مستقیم مغز بوسیله تحریک الکتریکی فراجمجمه ای^[[16]](#footnote-17)^ یک روش غیر تهاجمی است که یک جریان مستقیم نسبتآ ضعیف از طریق الکترودهای کوچک روی جمجمه به کورتکس مغز منتقل شده و باعث تعدیل تحریک پذیری کورتیکال با توجه به قطبیت تحریک می شود (35). tDCS آنودال سبب متعادل کردن پتانسیل غشای نورون و در نهایت باعث افزایش تحریک پذیری مدارهای نورونی شده؛ در حالی که tDCS کاتدال باعث کاهش تحریک پذیری شبکه های عصبی می شود (21, 36). این یک روش ایمن و بی درد می باشد. شواهد گذشته نشان می دهد که تحریک کورتکس حرکتی(M1) و حسی (S1) ، می تواند در کاهش درد تاثیر گذار باشد(6, 34, 37). همچنین یک مطالعه نشان داده است که تحریک هم زمان کورتکسM1 و S1 نسبت به تحریک M1 به تنهایی، در کاهش درد موثرتر است. tDCS آنودال می تواند سبب افزایش تحریک پذیری هم به صورت موضعی در منطقه تحریک و هم به واسطه ارتباطات بین نورونی در نقاط دورتر از منطقه تحریک شود (21).

در این راستا یک متاآنالیز نشان داده است که ترکیب ورزش با tDCS آنودال روی M1 می تواند در کاهش درد مزمن موثر باشد(6). همچنین یافته های مطالعه دیگر بیانگر آن است که استفاده هم زمان از تمرینات پاسچرال و tDCS آنودال روی M1 در افراد مبتلا به کمردرد مزمن، می تواند سبب کاهش درد بلافاصله بعد از درمان شود که حتی تا یک ماه پس از آن نیز ماندگار است. علاوه بر آن، در این مطالعه در ارزیابی تعادل توسط تست های بالینی، نیز بهبودی مشاهده شد (38). به عبارتی نتایج این مطالعه بیانگر این بودکه اعمال جریان tDCS آنودال همراه با تمرین درمانی پاسچرال در بهبود تعادل، ثبات و درد در بیماران کمردرد دارای اختلال پاسچرال مؤثر است و دو هفته تمرین پاسچرال به تنهایی قادر به بهبود اختلالات پاسچرال در بیماران مبتلا به کمردرد نیست. در این راستا مطالعه Sofia Straudi بر روی افراد مبتلا به کمردرد مزمن غیراختصاصی نیز نشان داد که تفاوت معناداری در میزان شدت درد و پرسشنامه سلامت بیماران یک ماه پس از ترکیب tDCS با تمرین درمانی گروهی وجود دارد (39). Adam Louis Ouellette و همکارانش نیز پروتکل یک مطالعه کارآزمایی بالینی دو سویه کور را انتشار داده اند، که هدف، مقایسه دو گروه tDCS واقعی به همراه تمرینات حسی - حرکتی و tDCS شم به همراه تمرینات حسی - حرکتی می باشد. در این مطالعه امکان پذیری، امنیت، درد، ناتوانی و عملکرد فرد قبل و پس از پایان ده هفته درمانارزیابی شده و در نهایت هدف از مطالعه بررسی اثرگذاری این پروتکل درمانی می باشد (31). اما با وجود اهمیت کنترل حسی- حرکتی کمر در افراد مبتلا به کمردرد، مطالعه ای در این زمینه انجام نشده است. همان طور که قبلا اشاره شد، مناطق حسی اولیه (S1) و حرکتی اولیه (M1) در افراد مبتلا به کمردرد دچار کاهش تحریک پذیری می شود. از این رو سوال مطالعه آن است که آیا افزایش تحریک پذیری این دو منطقه به وسیله tDCS آنودال و ترکیب آن با تمرینات حسی- حرکتی می تواند بر پردازش حسی و حرکتی مغز، کنترل حرکتی کمر و درد در بیماران مبتلا به کمردرد مزمن مؤثر باشد؟ و آیا بین بهبود پارامترهای نوروفیزیولوژیک مغزی و پارامترهای بالینی ارتباطی وجود دارد؟

## 2. هدف کلی

اثر ترکیب تمرینات حسی-حرکتی با تحریک الکتریکی فراجمجمه ای بر پردازش حسی- حرکتی مغز و علائم بالینی در افراد مبتلا به کمردرد مزمن

## 3. اهداف توصیفی

**اصلی (اولیه)**

- تعیین میانگین میزان کنترل حرکت کمر در گروه ها (مداخله و کنترل)، قبل و بعد از مداخله
- تعیین میانگین میزان ناتوانی در گروه ها (مداخله و کنترل)، قبل و بعد از مداخله
- تعیین میانگین میزان درد در گروه ها (مداخله و کنترل)، قبل و بعد از مداخله

**فرعی (ثانویه)**

- تعیین میانگین آمپلی تود N80 و N150در گروه ها (مداخله و کنترل)، قبل و بعد از مداخله
- تعیین میانگین آستانه حرکتی فعال عضله مالتی فیدوس و عرضی شکمی در گروه ها (مداخله و کنترل)، قبل و بعد از مداخله
- تعیین میانگین آمپلی تود پتانسیل برانگیخته حرکتی عضله مالتی فیدوس و عرضی شکمیدر گروه ها (مداخله و کنترل)، قبل و بعد از مداخله

## 4. اهداف تحلیلی

**اصلی (اولیه)**

- مقایسه میانگین میزان کنترل حرکت کمر در گروه ها (مداخله و کنترل)، بین قبل و بعد از مداخله
- مقایسه میانگین میزان کنترل حرکت کمر بین گروه ها (مداخله و کنترل) در قبل و بعد از مداخله
- مقایسه میانگین میزان ناتوانی در گروه ها (مداخله و کنترل)، بین قبل و بعد از مداخله
- مقایسه میانگین میزان ناتوانی بین گروه ها (مداخله و کنترل) در قبل و بعد از مداخله
- مقایسه میانگین میزان درد در گروه ها (مداخله و کنترل)، بین قبل و بعد از مداخله
- مقایسه میانگین میزان درد بین گروه ها (مداخله و کنترل) درقبل و بعد از مداخله

**فرعی (ثانویه)**

- مقایسه میانگین آمپلی تود N80 و N150در گروه ها (مداخله و کنترل)، بین قبل و بعد از مداخله
- مقایسه میانگین آمپلی تودN80 و N150 بین گروه ها (مداخله و کنترل) در قبل و بعد از مداخله
- مقایسه میانگین آستانه حرکتی فعال عضله مالتی فیدوس و عرضی شکمی در گروه ها (مداخله و کنترل)، بین قبل و بعد از مداخله
- مقایسه میانگین آستانه حرکتی فعال عضله مالتی فیدوس و عرضی شکمی بین گروه ها (مداخله و کنترل) درقبل و بعد از مداخله
- مقایسه میانگین آمپلی تود پتانسیل برانگیخته حرکتی عضله مالتی فیدوس و عرضی شکمی در گروه ها (مداخله و کنترل)، بین قبل و بعد از مداخله
- مقایسه میانگین آمپلی تود پتانسیل برانگیخته حرکتی عضله مالتی فیدوس و عرضی شکمی بین گروه ها (مداخله و کنترل) در قبل و بعد از مداخله
- بررسی ارتباط همبستگی بین پارامترهای بالینی و پارامترهای نوروفیزیولوژیک مغزی

## 5. سوالات و فرضیات پژوهشی

- آیا میانگین آمپلی تود N80 و N150در گروه ها (مداخله و کنترل) بین قبل و بعد از مداخله تفاوت دارد؟
- آیا میانگین آمپلی تود N80 و N150 بین گروه ها (مداخله و کنترل) در قبل و بعد از مداخله تفاوت دارد؟
- آیا میانگین آستانه حرکتی فعال عضله مالتی فیدوس و عرضی شکمی در گروه ها (مداخله و کنترل)،بین قبل و بعد از مداخله تفاوت دارد؟
- آیا میانگین آستانه حرکتی فعال عضله مالتی فیدوس و عرضی شکمی بین گروه ها (مداخله و کنترل) در قبل و بعد از مداخله تفاوت دارد؟
- آیا میانگین آمپلی تود پتانسیل برانگیخته حرکتی عضله مالتی فیدوس و عرضی شکمی در گروه ها (مداخله و کنترل)،بین قبل و بعد از مداخله تفاوت دارد؟
- آیا میانگین آمپلی تود پتانسیل برانگیخته حرکتی عضله مالتی فیدوس و عرضی شکمی بین گروه ها (مداخله و کنترل) در قبل و بعد از مداخله تفاوت دارد؟
- آیا میانگین میزان کنترل حرکت کمر در گروه ها (مداخله و کنترل)،بین قبل و بعد از مداخله تفاوت دارد؟
- آیا میانگین میزان کنترل حرکت کمر بین گروه ها (مداخله و کنترل) در قبل و بعد از مداخله تفاوت دارد؟
- آیا میانگین میزان ناتوانی در گروه ها (مداخله و کنترل)،بین قبل و بعد از مداخله تفاوت دارد؟
- آیا میانگینمیزان ناتوانی بین گروه ها (مداخله و کنترل) در قبل و بعد از مداخله تفاوت دارد؟
- آیا میانگین میزان درد در گروه ها (مداخله و کنترل)،بین قبل و بعد از مداخله تفاوت دارد؟
- آیا میانگین میزان درد بین گروه ها (مداخله و کنترل) درقبل و بعد از مداخله تفاوت دارد؟
- آیا بین پارامترهای بالینی و پارامترهای نوروفیزیولوژیک مغزی ارتباط معنادار وجود دارد؟

## 6. اهداف کاربردی

در صورتی که توأم کردن تمرینات با tDCS بتواند نتایج بهتری به ارمغان بیاورد، میتوان این روش را برای استفاده در کلینیک پیشنهاد داد. چنانچه گروه های درمانی تفاوت چندانی با هم نشان ندادند، میتوان پیشنهاد داد تمرینات به تنهایی کارآمد هستند و از صرف هزینه بیشتر جلوگیری شود یا به دنبال روش جدید درمانی خواهیم بود.

## 7. جنبه جدید بودن موضوع

بررسی مطالعات نشان می دهد که tDCS و تمرینات حسی – حرکتی میتوانند بر کاهش درد بیماران مبتلا به کمر درد مزمن تاثیر گذار باشد. اما همان طور که اشاره شد در این بیماران پردازش حسی و حرکتی مغز دچار اختلال می شود. این یافته ها در راستای دیگر شواهد رفتاری است که اذعان دارند در کنترل حسی- حرکتی کمر در این بیماران اشکالاتی وجود دارد که در نهایت می تواند باعث ناتوانی این افراد شود. آن چنان که کنترل حسی- حرکتی به عنوان یکی از فاکتورهای کلیدی ممکن در پیشرفت و رجوع کمردرد شناخته می شود. اما علیرغم اهمیت تغییرات مغزی در مناطق حسی و حرکتی و اختلال در کنترل حسی- حرکتی کمر در افراد مبتلا به کمردرد، تاکنون هیچ مطالعه ای در جهت بررسی اثر جریان tDCS آنودال روی مناطق حسی و حرکتی وترکیب آن با تمرینات حسی – حرکتی بر پردازش مغزی و بهبود کنترل حسی- حرکتی کمر در این بیماران صورت نگرفته است. درپژوهشحاضر برایاولینبارتاثیر جریان tDCS آنودال بر منطقه M1 و S1 به همراه تمرينات، بر بهبود پارامترهای مذکور سنجیدهخواهدشد. از این رو هدف در این مطالعه پی بردن به این سوال است که با توجه به کاهش تحریک پذیری مناطق M1 و S1 در افراد مبتلا به کمردرد مزمن، آیا تحریک این مناطق بوسیله tDCS آنودال و همراه کردن آن با تمرینات، میتواند درمان مؤثرتری برای این افراد باشد یا نه. در این مطالعه علاوه بر پارامترهای بالینی، تمرکز بر پارامترهای نوروفیزیولوژیکال هم است. از این رو یکی از نتایج مطالعه پی بردن به مکانسیم های احتمالی است و در واقع میتوان به ارتباط بین بهبود پارامترهای بالینی و تغییر پارامترهای نوروفیزیولوژیکال به دنبال درمان پی برد که پیشتر به آن پرداخته نشده است.

**8. بررسی مطالعات پیشین**

در این قسمت مقالات پیشین در چند زیر مجموعه بررسی میشود:

- اثر ترکیب تحریک الکتریکی فراجمجمه ای و تمرینات حسی-حرکتی
- اثر ترکیب تحریک الکتریکی فراجمجمه ای و سایر تمرینات
- اثرتحریک الکتریکی فراجمجمه ای بر درد
- اثر تمرینات حسی-حرکتی بر سازماندهی مجدد مغز
- اثر تمرینات حسی-حرکتی بر روی درد و عملکرد عضلانی

##

## اثر ترکیب تحریک الکتریکی فراجمجمه ای و تمرینات حسی-حرکتی

- Adam Louis Ouellette و همکارانش در سال 2017، پروتکل یک مطالعه کارآزمایی بالینی دو سویه کور را انتشار داده اند. در این مطالعه پیش بینی شده است که 80 بیمار مبتلا به کمردرد مزمن به صورت تصادفی به دو گروه tDCS واقعی + تمرینات حسی – حرکتی و tDCS شم + تمرینات حسی – حرکتی تقسیم شوند. tDCS به مدت 20 دقیقه و بر روی کورتکس حرکتی اولیه به همراه 60 دقیقه تمرینات حسی – حرکتی به مدت دو بار در هفته و در کل به مدت 10 هفته اعمال می شود. در این مطالعه امکان پذیری^[[17]](#footnote-18)^ (شامل تعداد جلسات درمانی هر بیمار، میزان ریزش بیماران در هرگروه، نسبت تعداد افراد شرکت کننده از کل تعداد افرادی که غربالگری شده اند، میزان علاقه مندی افراد به ادامه درمان و تعداد جلسات تمرین در منزل تکمیل شده)، امنیت، درد، ناتوانی و عملکرد فرد قبل و پس از پایان ده هفته درمان ارزیابی شده و در نهایت هدف از مطالعه بررسی اثرگذاری^[[18]](#footnote-19)^ این پروتکل درمانی می باشد (31).

## اثر ترکیب تحریک الکتریکی فراجمجمه ای و سایر تمرینات

- Jafarzadeh و همکاران در سال 2019 تاثیر کوتاه مدت و بلند مدت ترکیب جریان tDCS آنودال و تمرینات پاسچرال را در بیماران مبتلا به کمردرد که اختلال پاسچرال هم داشتند، بررسی کردند. 38 فرد در سه گروه (tDCS آنودال واقعی به همراه تمرینات، tDCS آنودال شم به همراه تمرینات و گروه تمرینات به تنهایی) قرار گرفتند. همه ی افراد سه مرتبه در هفته به اندازه 20 دقیقه و به مدت دو هفته تمرینات پاسچرال را دریافت کردند. جریان آنودال با شدت 2 میلی آمپر و به مدت 20 دقیقه روی ناحیهM1 اعمال شد. قبل، بلافاصله و یک ماه بعد از انجام مداخله، متغیر های ثبات پاسچرال، تعادل و درد اندازه گیری شد. هر سه متغیر به طور معناداری در گروه آنودال همراه با تمرین درمانی بلافاصله و بعد از یک ماه بهبود یافتند و هیچ تفاوتی در سایر گروه ها مشاهده نشد. نتایج این مطالعه بیانگر این است که اعمال جریان tDCS آنودال همراه با تمرین درمانی پاسچرال در بهبود تعادل، ثبات و درد در بیماران کمردرد دارای اختلال پاسچرال مؤثر است و دو هفته تمرین پاسچرال به تنهایی قادر به بهبود اختلالات پاسچرال در بیماران مبتلا به کمردرد نیست (38).
- یک کارآزمایی بالینی دو سویه کور به صورت پایلوت توسط Sofia Straudi و همکارانش در سال 2018، انجام شده است. در این مطالعه 35 بیمار مبتلا به کمردرد مزمن غیراختصاصی 5 جلسه تحریک مغزی به صورت شم یا واقعی را همراه با ده جلسه تمرین گروهی دریافت می کردند. پس از ارزیابی VAS، پرسشنامه Rolland Morris، EuroQuol-5 و پرسشنامه سلامت بیماران، نتیجه گیری شده است که تفاوت معناداری در میزان شدت درد و پرسشنامه سلامت بیماران یک ماه پس از درمان ترکیبی وجود دارد (39).
- یک مطالعه پروتکل که ترکیب tDCS با تمرین درمانی را می سنجد، توسط Cavalcante و همکارانش در سال 2020، منتشر شده است. آنها پیش بینی کرده اند که 60 بیمار مبتلا به کمردرد مزمن طی 4 هفته و12 جلسه، درمان ترکیبی (1-tDCS واقعی + تمرینات و یا 2- tDCSشم + تمرینات) را دریافت کنند. قبل درمان، بعد از درمان، 3 ماه و 6 ماه بعد از درمان، شدت درد، جنبه های عاطفی و حسی درد، عملکرد فیزیکال، ترس از حرکت مورد بررسی قرار می گیرد. تمرینات بصورت کششی، تقویتی و تمرینات کنترل حرکتی بودند (40).
- در یک مطالعه پایلوت و دو سویه کور که توسطYuanbo Ma و همکارانش در سال 2020، انجام شد، تاثیر high definition tDCS و تمرینات تقویتی کف پا بر روی حس عمقیِ و تعادل داینامیک در افراد مبتلا به بی ثباتی مزمن مچ پا مورد بررسی قرار گرفت. در این مطالعه 30 فرد بزرگسال به مدت چهار هفته تحت درمان قرار گرفتند و تحریک tDCS در جهت تسهیل تحریک پذیری ناحیه M1 و S1 بود. در انتهای مطالعه اینطور مشاهده شد که ترکیب این دو درمان سبب بهبود تعادل داینامیک و حس عمقی در این افراد می شود (41).
- در مطالعه ای شم-کنترل که توسط Mendonca و همکارانشدر سال 2016، با هدف تعدیل سیستم حرکتی در افراد مبتلا به فیبرومیالژیا انجام شد، tDCSرا بر روی M1 قرار داده و با تمرینات ایروبیک ترکیب کردند. 45 بیمار در مطالعه شرکت کردهکه به سه گروه تقسیم شدند (1- تمرین + tDCS،2- فقط تمرین 3- فقط tDCS). متغیر های وابسته شامل شدت درد، میزان عصبانیت، کیفیت زندگی، حالات روحی فرد، آستانه درد فشاری و پلاستیسیتی کورتیکال بود. در پایان مشاهده شد که این سه گروه هیچ تفاوت چندانی در میزان پلاستیسیتی کورتیکال نشان نداده اند. ترکیب این دو می تواند تاثیرات زیادی بر روی درد، میزان عصبانیت و حالات روحی بگذارد. در نتیجه به لحاظ پلاستیسیتی بر سایر مدارهای نورونی مثل مدارهایی که به کنترل جنبه های عاطفی-احساسی درد مربوط اند، تاثیر می گذارد (42).

## اثرتحریک الکتریکی فراجمجمه ای بر درد

- در پژوهشی دو سویه کور که توسط Fuad Ahmad Hazimeو همکارانش در سال 2017، انجام شد، 92 بیمار مبتلا به کمردرد غیر اختصاصی مزمن به چهار گروه (1-tDCS واقعی + تحریک محیطی واقعی؛2- tDCS واقعی + تحریک محیطی شم؛3-tDCS شم + تحریک محیطی واقعی؛4-tDCS شم + تحریک محیطی شم) تقسیم شدند. جلسات درمانی درمدت 4 هفته و هفته ای 3 جلسه (مجموعا 12 جلسه) انجام گرفت. متغیر اولیه درد، قبل جلسه، در طی جلسه و در پایان جلسه مورد ارزیابی قرار گرفت. tDCS با شدت 2 میلی آمپر و به مدت 20 دقیقه اعمال شد، به گونه ای که الکترود فعال بر روی ناحیه C3 و C4 در سمت مخالف درد و الکترود غیر فعال بر روی ناحیه سوپرااوربیتال قرار داشت. در گروه تحریک محیطی جریان به صورت دو فازی غیر قرینه و مستطیلی شکل با فرکانس 100 هرتز و مدت زمان پالس 200 میکروثانیه و به مدت 40 دقیقه بر روی دردناک ترین قسمت کمر اعمال شد. پس از ارزیابی، محققین به این نتیجه دست یافتند که استفاده از tDCS + تحریک محیطی در بلند مدت و تحریک محیطی به تنهایی در کوتاه مدت می تواند در کاهش کمردرد موثر باشد ولی tDCS به تنهایی نقش موثری در کاهش درد ندارد (20).
- در پژوهشی دوسویه کور که توسط Timothy Y. Mariano و همکارانش در سال 2018، انجام شد، 30 بیمار مبتلا به کمردرد مزمن به دو گروه پلاسبو و کنترل تقسیم شدند. در طی ده جلسه درمانی tDCS کاتدال با شدت 2 میلی آمپر و به مدت 20 دقیقه بر روی ناحیه FC_1_اعمال شد. پس از اتمام جلسات مشاهده شد که درد و افسردگی بهبود پیدا کرده است(43).

## اثر تمرینات حسی-حرکتی بر سازماندهی مجدد مغز

- Bae و همکارانش پژوهشی بر روی 14 فرد (7 نفر سالم و 7 نفر مبتلا به کمردرد مزمن) در سال 2014، انجام داده اند. هدف از این مطالعه بررسی اثر تمرینات حسی – حرکتی بر تنظیمات پیش بینانه کنترل پوسچر^[[19]](#footnote-20)^، تغییرات مغزی در مناطق مربوط به کورتکس حرکتی توسط ثبت الکتروانسفالوگرافی، زمان شروع^[[20]](#footnote-21)^ انقباض عضلات عرضی شکمی^[[21]](#footnote-22)^ و مورب خارجی^[[22]](#footnote-23)^ بوسیله ثبت الکترومیوگرافی و همچنین درد بوده است. در این مطالعه افراد مبتلا به کمردرد مزمن در گروه تجربی جای گرفته و به مدت 4 هفته و هفته ای 4 جلسه تمرینات را انجام دادند. در انتهای جلسات تغییرات معناداری در پارامترهای مربوط به ثبت الکتروانسفالوگرافی از جمله پتانسیل آمادگی^[[23]](#footnote-24)^، پتانسیل حرکتی^[[24]](#footnote-25)^ و پتانسیل نظارت بر حرکت^[[25]](#footnote-26)^ مشاهده شد. بر طبق نتایج حاصل از بررسی مناطق مربوط به کورتکس حرکتی مشخص شد که پتانسیل آمادگی و پتاسیل حرکتی در مناطق مربوطه کاهش یافته است و همچنین در شروع انقباض عضلات عرضی شکمی و مورب خارجی شکمی تفاوت معناداری مشاهده شد. در هر دو عضله این زمان کاهش یافت ولی این میزان کاهش در عضله مورب خارجی بیشتر بود. علاوه بر آن کاهش چشمگیری در میزان درد حاصل شد(33).
- در پژوهشی که توسط Rocco Cavaleri و همکارانش در سال 2020 ، انجام شده است،30 فرد سالم به صورت تصادفی به دو گروه تقسیم شدند. این افراد در یک جلسه تمرین بینایی-حرکتی^[[26]](#footnote-27)^ شرکت کردند. جلسه درمانی به سه فاز (پایه، آزمایش و ریکاوری) تقسیم شد. در فاز پایه دو مرتبه ارزیابی تظاهرات کورتیکوموتور عضله ارکتوراسپاینال انجام گرفت. در فاز آزمایش یکی از دو تمرین مورد نظر که شامل تیلت لومبوپلویک در گروه آزمایش و ابداکشن انگشت تکراری در گروه کنترل به مدت 15 دقیقه بود انجام گرفت. در این فاز هم تظاهرات کورتیکوموتور عضله ارکتور اسپاینال جهت ارزیابی نقشه مغزی(با استفاده از TMS) مورد بررسی قرار گرفت. در فاز ریکاوری در 15 و 30 دقیقه پس از پایان تمرینات ماندگاری هر گونه تطابق کورتیکوموتور مورد بررسی قرار گرفت. در انتهای مطالعه مشاهده شد که هیچ ارتباط معناداری بین سازماندهی کورتیکوموتور و پیشرفت در اجرای تمرینات وجود ندارد و تغییرات بیشتر مربوط به مناطق ساب کورتیکال و یا شبکه های نخاعی است تا اینکه مربوط به تطابق در راه های کورتیکوموتور باشد. اگرچه محققین معتقدند پژوهش های بیشتر در حیطه انجام فعالیت هایی با پیچیدگی ها و مدت زمان های متفاوت جهت تایید این فرضیه مورد نیاز است (44).

## اثر تمرینات حسی-حرکتی بر روی درد و عملکرد عضلانی

- در پژوهشی که توسط مریم نظرزاده ده بزرگی و همکارانش در سال 2015، انجام شد،53 بیمار مبتلا به کمردرد غیراختصاصی مزمن به دو گروه کنترل و آزمایش تقسیم شدند. تمرینات حسی – حرکتی با استفاده از دستگاه هوبر به مدت 5 هفته و هفته ای دو جلسه 30 دقیقه ای انجام شد. بهبودی معناداری در کنترل حرکتی کمر (آزمون های کنترل حرکت لوماجوکی^[[27]](#footnote-28)^) در گروه تمرینات حسی – حرکتی مشاهده شد. همچنین کاهش معناداری در میزان درد این گروه نسبت به گروه کنترل مشاهده شد (29).
- در یک پژوهش که توسط Michael A. McCaskey و همکارانش در سال 2018 ، انجام شد،22 بیمار با کمردرد مزمن به طور تصادفی به دو گروه آزمایش و کنترل تقسم شدند. هر دو گروه درمانهای روتین فیزیوتراپی را به مدت 30 دقیقه دریافت می کردند اما در گروه آزمایش 15 دقیقه تمرینات پاسچرال حسی – حرکتی و گروه کنترل 15 دقیقه تمرینات قلبی عروقی با شدت کم و با تاثیر پایین انجام دادند. متغیرهای اولیه درد و عملکرد فرد از طریق Oswestry Disability Index بود. در نهایت مشاهده شد که تمرینات پاسچرال حسی – حرکتی تاثیر چشمگیری بر عملکرد فرد دارد اما هیچ تاثیر قابل توجهی بر کاهش درد یا بهبود عملکرد دو گروه با شدت درد متوسط ندارد (45).
- Jin Ah hwang و همکارانش در سال 2013، پژوهشی انجام دادند که هدف از آن بررسی اثر تمرینات حسی – حرکتی بر روی تنظیمات پیش بینانه کنترل پوسچر افراد مبتلا به کمردرد مزمن بود. این مطالعه سه گروه داشت؛ دو گروه آزمایشی که یک گروه تمرینات معمولی فیزیوتراپی و گروه دیگر تمرینات حسی – حرکتی را دریافت می کردند و دیگری گروه کنترل بود. دو گروه آزمایش تمرینات مربوط به خود را به مدت 40 دقیقه و 5 جلسه در هفته و در کل به مدت 4 هفته انجام می دادند. متغیر های اولیه درد، عملکرد فرد از طریق Oswestry Disability Index و زمان شروع انقباض عضلات عرضی شکمی و مورب خارجی با استفاده از ثبت الکترومیوگرافی بود. در بررسی های نهایی تغییرات چشمگیری در درد و مقیاس ناتوانی مشاهده شد. همچنین زمان شروع انقباض عضلات ذکر شده هم در وضعیت نشسته و هم در وضعیت ایستاده بسیار کاهش یافت. در نهایت تمرینات حسی – حرکتی به بیماران یاد می دهد که چگونه عضلات خود را تنظیم کنند و متعاقب با آن کاهش درد و بهبود عملکرد فرد حاصل می شود (46).
- در یک پژوهش که توسط Nisha Kanabarدر سال 2016 ، انجام شد، به بررسی میزان تغییر درد و عملکرد فرد از طریق Oswestry Disability Index پرداخته شد. در این مطالعه بیماران به دو گروه درمانی تمرینات حسی – حرکتی (13 نفر) و تمرینات ثباتی^[[28]](#footnote-29)^ (15 نفر) تقسیم شدند. هر گروه تمرینات را به مدت 4 هفته و هفته ای 5 جلسه انجام دادند. این پژوهش نشان داده است که هر دو نوع تمرین حسی – حرکتی و ثباتی می تواند در کاهش درد و بهبود عملکرد در بیماران مبتلا به کمردرد غیراختصاصی مزمن موثر باشد اگرچه تمرینات حسی – حرکتی تاثیرات بهتری نشان دادند (30).
- Benedict M. Wandو همکارانش در سال 2011، پژوهشی مقدماتی و پایه را با تنها سه بیمار مبتلا به کمردرد غیراختصاصی مزمن انجام دادند. هدف از این مطالعه بررسی اثر تمرینات حسی – حرکتی درجه بندی شده^[[29]](#footnote-30)^ بر شدت درد، تداخل درد^[[30]](#footnote-31)^ و زندگی روزانه، ناتوانی و ارزیابی امنیت^[[31]](#footnote-32)^ (با استفاده از ثبت هر گونه واکنش های مضر) برنامه بود. پس از ارزیابی های لازم به این نتیجه رسیدند که میزان درد، تداخل درد و میزان ناتوانی کاهش یافته است و هیچ گونه اثر سوء از انجام تمرینات گزارش نشده است (7).
- در یک آزمایش تک سویه که توسط Pia-Maria Wippert و همکارانش در سال 2019، انجام شد، 439 داوطلب که به صورت تصادفی به سه گروه تقسیم شدند، شرکت داشتند. این آزمایش طی 12 هفته (3 هفته در مرکز و 9 هفته در خانه) انجام شد. در این مطالعه میزان تغییرات درد، ناتوانی و سلامت روانی افراد مورد بررسی قرار گرفت. در گروه اول افراد تنها تمرینات حسی – حرکتی را دریافت کردند. در گروه دوم تمرینات حسی – حرکتی به همراه تمرینات مربوط به رفتار درمانی دریافت شد و گروه سوم به عنوان گروه کنترل صرفا تمرینات روتین سرپایی را انجام دادند. نسبت به روش های روتین، دیده شد که درمان ها تاثیرات قابل توجهی در میزان درد و ناتوانی دارند. همچنین، بهبود بسیاری در سلامت روانی افراد مشاهده شد. همچنین نسبت به روش های معمول این روش بسیار کم هزینه تر بود (47).

**نکات:** بررسی مطالعات نشان می دهد که tDCS و تمرینات حسی – حرکتی میتوانند بر کاهش درد بیماران مبتلا به کمر درد مزمن تاثیر گذار باشد. اما همان طور که اشاره شد در این بیماران پردازش حسی و حرکتی مغز دچار اختلال می شود. این یافته ها در راستای دیگر شواهد رفتاری است که اذعان دارند در کنترل حسی- حرکتی کمر در این بیماران اشکالاتی وجود دارد که در نهایت می تواند باعث ناتوانی این افراد شود. آن چنان که کنترل حسی- حرکتی به عنوان یکی از فاکتورهای کلیدی ممکن در پیشرفت و رجوع کمردرد شناخته می شود. اما علیرغم اهمیت تغییرات مغزی در مناطق حسی و حرکتی و اختلال در کنترل حسی- حرکتی کمر در افراد مبتلا به کمردرد، تاکنون هیچ مطالعه ای در جهت بررسی اثر جریانtDCS آنودال روی مناطق حسی و حرکتی و ترکیب آن با تمرینات حسی – حرکتی بر پردازش مغزی و بهبود کنترل حسی- حرکتی کمر در این بیماران صورت نگرفته است.

## 9. نوع مطالعه

کارآزمایی بالینی تصادفی یک سویه کور

## 10. جمعیت مورد مطالعه

جامعه هدف: بیماران مبتلا به کمردرد مزمن

جامعه مورد مطالعه: بیماران مبتلا به کمردرد مزمن در دسترس

## 11. روش نمونه گیری

نمونه ها به صورت نمونه گیری غیر احتمالی ساده، داوطلبانه و براساس ضوابط ورود و خروج انتخاب خواهند شد.

## 12. زمان و مکان انجام مطالعه

از اسفند 1400 تا تیر 1402

دانشکده توانبخشی دانشگاه علوم پزشکی تهران – آزمایشگاه بیومکانیک و همچنین مرکز نقشه برداری مغز ایران

## 13. تعداد حجم نمونه

حجم نمونه با توجه به مطالعه جعفرزاده و براساس متغیر درد و با کمک نرم افزارG*Power 3.1.3 محاسبه شد. در این محاسبه از f کوهن بین گروهی 473/0، توان 8/0 و آلفای 05/0 استفاده شد. این تحلیل توان نشان داد که حداقل 30 شرکت کننده مورد نیاز هستند. از این رو با احتساب احتمال ریزش 15 درصد، 34 فرد شرکت کننده برای مطالعه پیش بینی شد.

## 14. معیارهای ورود به مطالعه

- بازه سنی بین 20 تا 50 سال
- هر دو جنس زن و مرد (39)
- داشتن کمردرد به مدت بیش از 6 ماه یا 3 دوره کمردرد به مدت بیش از یک هفته در طول 12 ماه گذشته (38)
- درد رادیکولار یک طرفه ثانویه به فتق دیسک L4/L5 و L5/S1تشخیص داده شده با MRI(48)
- مثبت شدن حداقل یکی از تست های Slump ، Straight Leg Raise ، Lasegues’s sign(48)
- مسیر انتشار درد از قدامی خلفی ساق تا ناحیه پشت پا مرتبط با درماتوم L4/L5 تا خلف ساق تا پاشنه و قسمت خارجی پا (48)
- متوسط شدتدرد می بایست 4 یا بیشتر طبق مقیاس عددی درد باشد(31).
- متوسط ناتوانی فرد براساس Oswestry Disability Index می بایست 4 یا بیشتر باشد.
- نداشتن تومورهای نخاعی (31)
- نداشتن اختلالات شناختی طبق مقیاس Mini Mental Status Examination ≥  (39)
- نداشتن اسپوندیلولیستزیس(30, 31) و اسپوندیلولیزیس(30)
- نداشتن اختلالات ساختاری یا دفورمیتی های ستون فقرات از جمله اسکولیوزیس، کایفوزیس، لوردوزیس شدید (38)
- نداشتن شکستگی های ستون فقرات (31)
- نداشتن آسیب های مغزی ازجمله ضربات مغزی و یا سکته مغزی
- نداشتن بیماری های نورولوژیکال مثل پارکینسون، آلزایمر، اختلالات مخچه ای (31, 38)
- نداشتن خراش یا بریدگی در ناحیه پوست جمجمه (31)
- نداشتن اختلال یا فقدان حسی (31)
- نداشتن سابقه صرع (42)
- نداشتن بارداری (40, 42)
- نداشتن کاشت شی فلزی در مغز (42)
- نداشتن عفونت های پوستی (30)
- نداشتن ضربان ساز یا سایر ابزارهای کاشت (40)
- نداشتن اختلال بینایی
- نداشتن افسردگی که فرد مجبور به مصرف دارو باشد
- نداشتن عمل جراحی ستون فقرات

## 15. معیارهای خروج از مطالعه

- عدم تمایل آزمودنی به انجام یا ادامه آزمایش (29)
- غیبت در دو جلسه متوالی و سه جلسه غیر متوالی از جلسات درمانی (29)
- اگر بیماری تحت یک برنامه خاص تمرینی برای کمردرد خود باشد (31).
- استفاده از مواد مخدر، داروهای آرامبخش یا ضد درد (42)
- در صورت مشاهده تحریک یا حساسیت پوست ناحیه سر در طی جلسات

## 16. مشخصات ابزارهای جمع آوری اطلاعات

1. ثبت پتانسیل برانگیخته حسی توسط دستگاه EMG/NCV/EP5000 Q
2. ثبت پتانسیل برانگیخته حرکتی توسط دستگاهTMSمدل MagPro X100
3. بررسی میزان کنترل حرکتی کمر توسط تست های بالینی Luomajoki
4. اندازه گیری میزان ناتوانی با نسخه فارسی پرسشنامه Aswestry Disability Index(پیوست 3)
5. اندازه گیری درد با شاخص Visual analog Scale (VAS) (پیوست 1)
6. بررسی مشکلات شناختی فرد با Mini mental status exam (MMSE)(پیوست 2)
7. ثبت خصوصیات دموگرافیک و بالینی بیمار با پرسشنامه طراحی شده (پیوست 1)
8. فرم رضایت نامه (پیوست 4)

## 17. روش کار

ابتدا مطالعه، کد اخلاق دریافت خواهد کرد و سپس در سامانه IRCT ثبت و کد کارآزمایی بالینی دریافت میکند. به شرکت‌کنندگانی که شرایط لازم برای شرکت در مطالعه را احراز خواهند کرد، توضیحاتی درمورد نحوه انجام تحقیق داده خواهد شد تا فرد شرکت‌کننده آگاهی کاملی در مورد چگونگی انجام مراحل داشته باشد. هر شرکت‌کننده موافقت آگاهانه خود را به صورت امضای رضایت‌نامه کتبی که مورد تأیید کمیته اخلاق دانشگاه علوم پزشکی تهران قرار گرفته است اعلام خواهد نمود. ابتدا ارزیابی‌ها انجام خواهد شد و درمان‌ها در گروه کنترل (گروه تمرینات حسی-حرکتی همراه با تحریک الکتریکی فراجمجمه‌ای شم) و در گروه مداخله (تمرینات حسی-حرکتی همراه با تحریک الکتریکی فراجمجمه‌ای واقعی) به مدت 4 هفته انجام خواهد شد و 24 تا 48 ساعت پس از آخرین جلسه درمان، ارزیابی مجدد صورت خواهد گرفت.

**17-1. ارزیابی**

ارزیابی‌ها قبل از شروع جلسه اول درمانی و 24 تا 48 ساعت بعد از اتمام درمان انجام خواهند شد. دو تست آزمایشگاهی و یک تست بالینی انجام خواهد گرفت و دو پرسش‌نامه نیز تکمیل خواهد گردید.

**17-1-1. ارزیابی‌های آزمایشگاهی**

ارزیابی‌های آزمایشگاهی شامل دو آزمایش خواهد بود، یکی به منظور بررسی پردازش حسی مغز و دیگری پردازش حرکتی مغز.

**17-1-1-1. آزمایش برای پردازش حسی مغز**

به منظور بررسی پردازش حسی مغز از پتانسیل برانگیخته حسی و از دستگاه EMG/NCV/EP5000 Q استفاده خواهد شد. به طور کلی هدف پتانسیل برانگیخته حسی، بررسی سیستم عصبی حسی است که با استفاده از اعمال تحریک سوماتوسنسوری باعث ثبت موج‌هایی از مغز به وسیله الکترودهای الکتروانسفالوگرافی خواهد شد (8). الکتروانسفالوگرافی منعکس‌کننده فعالیت الکتریکال خودبخودی مغز در طی یک مدت زمان کوتاه است، در صورتی که پتانسیل برانگیخته حسی بطور مداوم و خودبخودی ثبت نمی‌شود بلکه با یک پیش‌تحریک همراه است (50).

در این آزمایش دو متغیر (آمپلی‌تود N80 و N150) مورد بررسی قرار خواهند گرفت (تصویر 1). تحقیقات الکتروفیزیولوژیک نشان می‌دهند که پتانسیل برانگیخته حسی ثبت شده به وسیله الکتروانسفالوگرافی، منعکس‌کننده پردازش آوران‌های حسی در مناطق مجزایی از قشر مغز هستند. تصور می‌شود که پنجره زمانی N80 پردازش در منطقه حسی اولیه (S1) را نشان خواهد داد، در حالی که پنجره زمانی N150 بیانگر پردازش در قشر حسی ثانویه (S2) است (19).

جهت انجام آزمایش، شرکت‌کنندگان راحت بر روی صندلی خواهند نشست به طوری که پاها روی زمین باشند و دست‌ها در حالت راحت قرار داده خواهند شد. از شرکت‌کنندگان خواسته خواهد شد که حین انجام آزمایش با چشمان بسته بنشینند ولی بیدار باشند. جهت ثبت پتانسیل برانگیخته حسی، از الکترودهای سطحی بر روی پوست جمجمه استفاده خواهد شد. این الکترود بر روی منطقه S1 که بر طبق سیستم 20-10 بین‌المللی نسبت به Cz، 3 سانتی‌متر خارج‌تر و 2 سانتی‌متر عقب‌تر هست و در سمت مخالف به محل درد شرکت‌کننده جای‌گذاری خواهد شد (19). پوست سر در محل ثبت نباید بیشتر از 5 کیلو اهم مقاومت داشته باشد. الکترود رفرنس در ناحیه Fz و الکترود زمین بر روی پیشانی قرار خواهد گرفت (50).

الکترودهای دو قطبی برای اعمال تحریک بر روی کمر، 3 سانتی‌متر خارج از زائده خاری L3 و در همان سمتی که بیمار بیشترین درد را داشت، قرار داده خواهند شد. محرک‌های الکتریکی دارای مدت زمان پالس 1 میلی‌ثانیه بوده و با فرکانس 2 هرتز اعمال خواهند شد. پهنای باند بر 1 تا 500 هرتز تنظیم خواهد شد. تحریک با شدت 1 میلی‌آمپر، 1 میلی‌آمپر افزایش خواهد یافت تا به آستانه ادراک برسد. سپس شدت تحریک بر روی سه برابر آستانه درک تنظیم خواهد شد. در صورتی که این شدت، درد را برمی‌انگیخت، شدت تحریک، 1 میلی‌آمپر، 1 میلی‌آمپر کاهش داده خواهد شد تا زمانی که محرک دیگر دردناک نباشد (19). تحریکات 500 بار اعمال خواهند شد و این پروسه دو بار تکرار خواهد شد که میانگین دو تکرار وارد آنالیز نهایی خواهد شد. حداکثر آمپلی‌تود پتانسیل برانگیخته حسی، زیر 10 میکروولت خواهد بود (50). آمپلی‌تود N80 در واقع بزرگترین قله در محدوده 40 تا 90 میلی‌ثانیه از شروع موج هست. آمپلی‌تود N150 نیز بزرگترین قله در محدوده 90 تا 180 میلی‌ثانیه از شروع موج خواهد بود که در مطالعه حاضر N80 و N150 بیشتر در محدوده زمانی 65 و 120 میلی‌ثانیه مشاهده شدند. همان‌طور که در تصویر 2 مشاهده می‌شود، حداکثر آمپلی‌تود در واقع اختلاف بین بالاترین قله و پایین‌ترین دره خواهد بود (19).


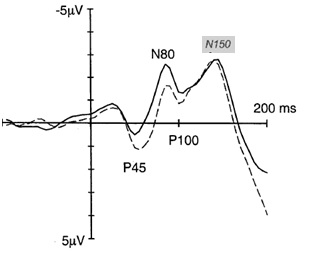


تصویر 1. ثبت پتانسیل برانگیخته حسی N80 و N150


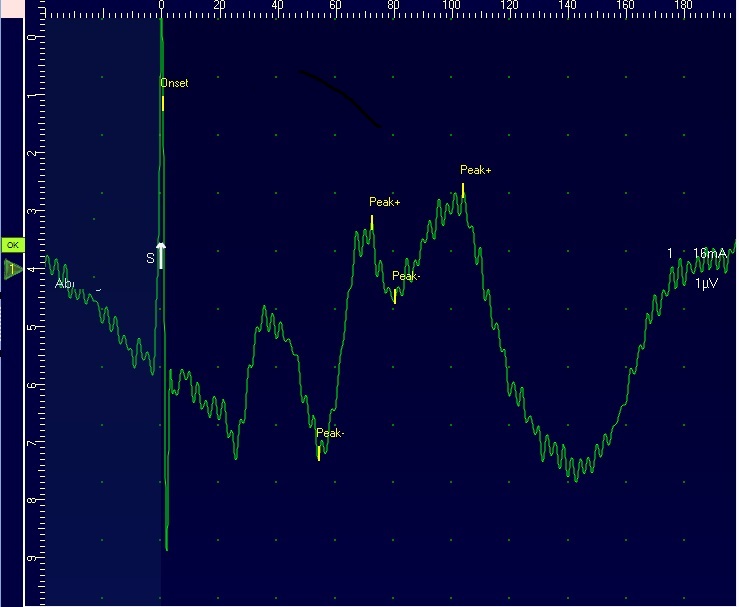


تصویر 2. نحوه محاسبه حداکثر آمپلی تود برای N80 و N150

#### 17-1-1-2. آزمایش برای پردازش حرکتی مغز

به منظور بررسی پردازش حرکتی مغز از آستانه حرکتی و پتانسیل برانگیخته حرکتی و دستگاه TMS مدل MagPro X100 استفاده خواهد شد. به طور کلی هدف پتانسیل برانگیخته حرکتی بررسی سیستم عصبی حرکتی از طریق اعمال یک تک پالس تحریکی به وسیله تحریک فراجمجمه‌ای مغناطیسی بر روی کورتکس حرکتی و سپس ثبت این تحریک حرکتی از عضله مربوطه به وسیله الکترودهای الکترومیوگرافی خواهد بود. موجی که ثبت خواهد شد، تحت عنوان پتانسیل برانگیخته حرکتی شناخته خواهد شد (11, 19, 34). آستانه حرکتی نیز شاخصی از تحریک‌پذیری کورتکس خواهد بود و همچنین به منظور تعیین میزان شدت تحریک به وسیله دستگاه استفاده خواهد شد.

در این آزمایش، 4 متغیر (آستانه حرکتی فعال و آمپلی‌تود پتانسیل برانگیخته حرکتی فعال عضله مالتی‌فیدوس و عرضی شکمی) مورد بررسی قرار خواهند گرفت. این متغیرها شاخصی از تحریک‌پذیری، عملکرد و یکپارچگی راه‌های عصبی و نواحی کورتیکواسپاینال سیستم حرکتی مربوط به این عضلات خواهند بود.

برای ثبت متغیرهای مربوط به عضله مالتی‌فیدوس، بیمار راحت روی صندلی خواهد نشست و پاهایش را بر روی زمین قرار خواهد داد. برای این عضله الکترود سطحی الکترومیوگرافی در سطح زائده خاری L5 و در راستای خطی که PSIS و فضای L1-L2 را به هم وصل کرده‌ایم قرار داده خواهد شد. الکترود زمین هم بر روی ایلیاک کرست قرار داده خواهد شد (23, 44). پهنای باند بر 5 تا 500 هرتز تنظیم خواهد شد. جهت ایجاد یک انقباض حداکثر در عضله مالتی‌فیدوس از فرد خواسته خواهد شد در برابر مقاومت دست اکستنشن تنه را انجام دهد و 3 ثانیه نگه دارد (44). این حرکت 3 بار تکرار خواهد شد و حداکثر انقباض براساس Root mean square (RMS) در یک پنجره 1 ثانیه به عنوان حداکثر انقباض لحاظ خواهد شد. سپس 20 درصد از آن به عنوان انقباض ساب ماگزیمال تعیین خواهد شد و در مانیتور این مقدار انقباض برای فرد نمایش داده خواهد شد تا در تمام طول ثبت این میزان انقباض حفظ شود (23, 44, 52, 53). این انقباض برای این است که ثبت پتانسیل برانگیخته حرکتی در عضلات پاراورتبرال آسان‌تر شود. به عبارتی در صورتی که عضله در وضعیت استراحت خود باشد، ثبت به سختی

صورت خواهد گرفت (23, 44).

برای ثبت متغیرهای مربوط به عضلات شکمی، بیمار راحت روی صندلی خواهد نشست و بازوها روی صندلی و زانو در حالت باز قرار خواهند گرفت. الکترود سطحی الکترومیوگرافی 2 سانتی‌متر پایین‌تر و 2 سانتی‌متر داخل‌تر نسبت به ASIS، که در واقع سطحی‌ترین بخش این عضلات خواهند بود، و الکترود زمین هم بر روی ایلیاک کرست قرار داده خواهند شد (54). پهنای باند بین 20 تا 450 هرتز تنظیم خواهد شد. جهت ایجاد یک انقباض حداکثر در عضلات شکمی از فرد خواسته خواهد شد که مانور بازدم اجباری را انجام دهد. این مانور به مدت 3 ثانیه حفظ و 3 بار تکرار خواهد شد. سپس 15 درصد از آن به عنوان انقباض ساب ماگزیمال لحاظ خواهد گردید و می‌بایست در تمام طول ثبت حفظ شود (52).

برای اعمال تحریک از کویل دابل مخروطی استفاده خواهد شد. این کویل برای تحریک قسمت حرکتی عضلات تنه ایده‌آل خواهد بود. این کویل در ناحیه M1 قرار داده خواهد شد، زیرا شواهد نشان داده‌اند که این نقطه بهترین نقطه جهت تحریک راه‌های کورتیکال و اعصاب حرکتی خواهد بود و در نتیجه پتانسیل برانگیخته بهتری می‌توان ثبت کرد. براساس مطالعات گذشته، کورتکس حرکتی مربوط به عضلات تنه 2 سانتی‌متر خارج‌تر نسبت به خط وسط و 2 سانتی‌متر جلوتر نسبت به ورتکس پیشنهاد خواهد شد. این تحریک در سمت مخالف بیشترین منطقه‌ای از کمر که درد دارد، اعمال خواهد شد (19, 34). کویل در زاویه 45 درجه نسبت به محور قدامی-خلفی قرار خواهد گرفت تا جریانی از عقب به جلو اعمال کند (19, 55).

در ابتدا آستانه حرکتی فعال تعیین خواهد شد. آستانه حرکتی فعال کمترین شدت TMS خواهد بود که باعث برانگیختن پتانسیل حرکتی با حداقل آمپلی تود 100 میکروولت در حداقل 5 تا از 10 تکرار خواهد شد. از آنجا که ثبت در هنگام انقباض ایزومتریک عضله صورت می‌گیرد، به آن فعال گفته خواهد شد. معمولاً شدت آغازین تحریک %25 حداکثر خروجی دستگاه تحریک در نظر گرفته خواهد شد و سپس تدریجا و به صورت %2، %2 شدت افزایش خواهد یافت تا ثبتی با خصوصیات گفته شده حاصل شود (56). فاصله بین سر و کویل نیز کمتر از 5 میلی‌متر در نظر گرفته خواهد شد (56).

پس از تعیین آستانه حرکتی فعال، شدت کویل بر روی %120 آستانه حرکتی فعال تنظیم شده و آمپلی تود پتانسیل برانگیخته حاصل نیز محاسبه خواهد گردید (26). آمپلی تود پتانسیل برانگیخته حرکتی فعال به عنوان RMS فعالیت الکترومیوگرافی عضلات از شروع تا پایان پتانسیل برانگیخته حرکتی تعریف خواهد شد (تصویر 3).


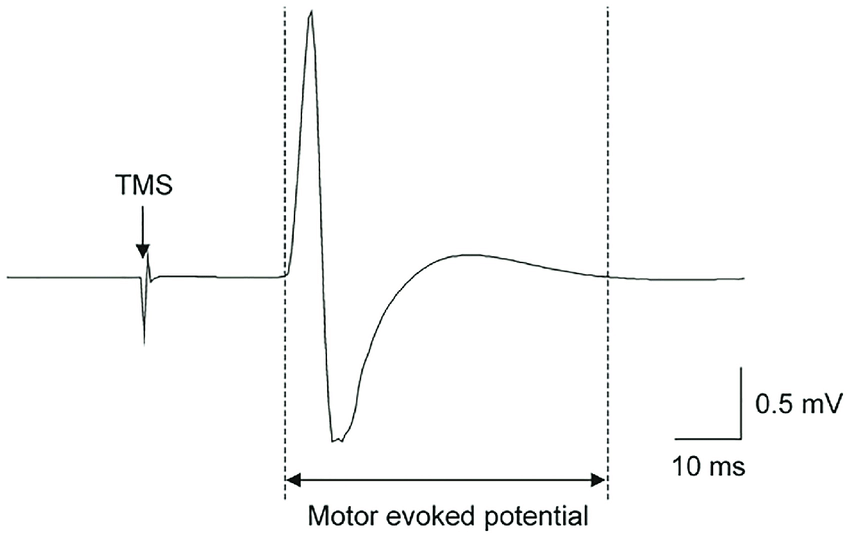


تصور 3. ثبت پتانسیل برانگیخته حرکتی

#### 17-1-2. ارزیابی های بالینی

#### 17-1-2-1. کنترل حرکتی کمر

به منظور ارزیابی کنترل حرکت کمر از 6 تست طراحی شده توسط Luomajokiکه دارای روایی بالای (6/0) هستند استفاده می شود. این تست ابزاری مطمئن برای تشخیص اختلال در کنترل لومبوپلویک است. ابتدا آزمونگر چگونگی انجام حرکت را توضیح میدهد و از فرد میخواهد حرکت را انجام دهد. اگر حرکت به درستی انجام نشود و یا فرد متوجه چگونگی انجام حرکت نشود با توضیحات سعی میشود که حرکت اصلاح شود. اگر در تکرار دوم باز حرکت به درستی انجام نشود، آزمونگر حرکت صحیح را به فرد نمایش میدهد. اگر فرد بعد از تکرار دوم مجددا نتواند حرکت را به درستی انجام دهد، نمره 1 و درصورت انجام صحیح، نمره 0 به آن تست تعلق میگیرد. نمره 0 به آن معنی است که همه تستها به درستی انجام شده و نمره 6 به معنای آن است که هیچ تستی درست انجام نشده است. به طور کلی هر حرکت 3 بار مجاز به انجام است (29, 50) (جدول 1).

جدول 1- تستهای کنترل حرکتی کمر

| **تست** | **توضیحات کلی** | **حرکت درست** | **حرکت نادرست** |
| --- | --- | --- | --- |
| **تست 1**  **“Waiters bow”** | فلکشن هیپ ها در ایستادن صاف بدون فلکشن و حرکت کمر | خم شدن هیپ ها بدون حرکت کمر (50-70 درجه فلکشن هیپ ها) | خم شدن هیپ ها کمتر ار 50 درجه و یا حرکت کمر |
| **تست 2**  **“Pelvic tilt”** | تیلت روبه عقب لگن در هنگام ایستادن صاف | ایستادن صاف، حفظ مهره های توراسیک در وضعیت نوترال و حرکت مهره های کمری به سمت فلکشن | لگن نمیتواند تیلت شود یا کمر به سمت اکستنشن میرود یا جبرانی مهره های توراسیک به فلکشن میرود. |
| **تست 3**  **“One leg stance”** | حرکت از حالت طبیعی ایستادنبه ایستادن روییک پا و اندازه گیری حرکت جانبیناف. | فاصله جابجایی در چپ و راست قرینه است. جابه جایی بیش از 2 سانتیمتر نیست. | جا به جایی طرفی بیش از 10 سانتیمتر و اختلاف دو سمت بیش از 2 سانتیمتر است. |
| **تست 4**  **" Sitting knee extension"** | نشستن با قوس کمری نوترال و اکستنشن زانو بدون حرکت و فلکشن کمر | نشستن با قوس کمری نوترال و اکستنشن زانو بدون حرکت و فلکشن کمر (30-35 درجه اکستنشن زانو نرمال است.) | کمر به سمت فلکشن میرود و بیمار از حرکت کمر آگاه نیست. |
| **تست 5**  **“Quadruped position”** | در حالت چهار دست و پا قرار گرفته و درحالیکه کمر در وضعیت نوترال قرار دارد لگن به سمت جلو و عقب برده میشود. وضعیت شروع 90 درجه فلکشن هیپ است. | 120 درجه فلکشن هیپ بدون حرکت کمر با جا به جایی لگن به سمت عقب | فلکشن هیپ باعث فلکشن کمر میشود و بیمار از حرکت کمر آگاه نیست. |
| **تست 6**  **" Prone lying active knee Flexion"** | بیمار روی شکم دراز کشیده و زانو را خم میکند. | فلکشن زانو حداقل 90 درجه بدون حرکت کمر و لگن | با فلکشن زانو کمر در حالت نوترال باقی نمی ماند بلکه به سمت اکستنشن و یا روتیشن میرود. |

#### 17-1-2-2. میزان ناتوانی

در ارزیابی ناتوانی افراد از نسخه فارسی پرسشنامه Oswestry Disability Indexاستفاده میشود. این پرسشنامه جهت ارزیابی ناتوانی عملکردی افراد مبتلا به کمردرد استفاده می شود که شامل ده بخش از جمله شدت درد، مراقبت های فردی، راه رفتن، نشستن و ... است که در هر بخش، هر فرد نمره ای بین 0تا 5دریافت می کند. در نهایت مجموع تمامی نمرات بصورت عدد محاسبه میشود. بگونه ای که نمره 4-0 نشان دهنده عدم ناتوانی، نمره 14-5 ناتوانی خفیف، نمره 15-24 ناتوانی متوسط، نمره 24-34 ناتوانی شدید و نمره 35-50 ناتوانی کامل میباشند (30).

#### 17-1-2-3. میزان درد

به منظور ارزیابی شدت درد از مقیاس بصری درد یا VASاستفاده میشود. در این مقیاس از یک نوار به اندازه 10 سانتیمتر استفاده میشود و از فرد خواسته میشود از یک تا ده به شدت درد خود نمره دهد. صفر نشان دهنده عدم درد و ده نشان دهنده درد غیر قابل تحمل است (48). این مقیاس کمی گسسته بوده و جهت بررسی تغییرات شدت درد بیمار قبل از اعمال مداخلات درمانی و پس از آن استفاده میشود.

### 17-2. درمان ها

پس از اتمام ارزیابی اولیه، افراد به طور تصادفی به دو گروه تقسیم خواهند شد. بدین ترتیب که توالی از طریق سایت randomization.com و به روش balanced block randomization تولید خواهد شد. سایز بلوک‌ها 4 تایی خواهد بود. همچنین به منظور allocation concealment از پاکت‌های مهر و موم شده و شماره‌گذاری شده استفاده خواهد شد که توسط منشی در اختیار افراد شرکت‌کننده قرار خواهد گرفت. دوره درمان در هر دو گروه 12 جلسه و به صورت 3 بار در هفته خواهد بود. بعد از پایان دوره درمانی، ارزیابی‌ها بار دیگر انجام خواهند شد.

به دلیل داشتن درمان شم، افراد شرکت‌کننده کورسازی خواهند شد اما امکان کورسازی درمانگر و یا ارزیابی‌کننده وجود نخواهد داشت.

17-2-1. گروه کنترل

در این گروه، افراد شرکت‌کننده تمرینات حسی- حرکتی را همراه با تحریک الکتریکی فراجمجمه‌ای شم به مدت 4 هفته و 3 بار در هفته (12 جلسه) دریافت خواهند کرد. شرکت‌کنندگان ابتدا جریان را دریافت خواهند کرد، سپس تمرینات را انجام خواهند داد.

17-2-2. گروه مداخله

در این گروه، افراد شرکت‌کننده تمرینات حسی- حرکتی را همراه با تحریک الکتریکی فراجمجمه‌ای آنودال به مدت 4 هفته و 3 بار در هفته (12 جلسه) دریافت خواهند کرد. شرکت‌کنندگان ابتدا جریان را دریافت خواهند کرد، سپس تمرینات را انجام خواهند داد.

17-2-3. تمرینات حسی- حرکتی

در تمرینات حسی- حرکتی، هدف افزایش ورودی‌های حس عمقی از سه منطقه (کف پا، مفصل ساکروایلیاک و مهره‌های گردنی) خواهد بود تا الگوهای حرکتی هماهنگ و اتوماتیک را تسهیل کند. از این رو در تمام مراحل انجام تمرینات، قرارگیری وضعیت صحیح این سه منطقه ضروری خواهد بود. برای تحریک گیرنده‌های کف پا، تمرینات به صورت پابرهنه انجام خواهند شد. سپس از فرد خواسته خواهد شد عضلات کف پا را منقبض کند طوری که قوس داخلی پا افزایش پیدا کرده ولی انگشتان پا خم نشوند. در ابتدا برای افرادی که قادر به انقباض عضلات کف پا نبودند، نواری از تیپ به کف پا چسبانده خواهد شد تا به صورت کمکی پا در وضعیت مناسب قرار گیرد.

در حین تمرینات، مفاصل ساکروایلیاک و مهره‌های گردنی هم بایستی در حالت نوترال قرار بگیرند. همچنین از فرد خواسته خواهد شد کمی ناف را به داخل بکشد تا عملکرد عضلات عرضی شکم تسهیل گردد. علاوه بر این، فرد با chin tuck باعث فعال شدن عضلات فلکسور عمقی گردن خواهد شد. به طور کلی تمرینات در دو مرحله (استاتیک و داینامیک) انجام خواهند گرفت.

مرحله استاتیک

در این مرحله تمرکز بر ثبات لگن بوسیله انقباض عضلات دیافراگم، مالتی فیدوس، کف لگن و عرضی شکم خواهد بود تا اساس برای انجام حرکات اندام‌ها در مراحل بعدی فراهم شود. به عبارتی این مرحله براساس اصل "ثبات پروگریمال برای حرکات دیستال" استوار خواهد بود. نحوه‌ی پیشروی در این مرحله از ایستادن روی دو پا، ایستادن بر روی تک پا و سپس ایستادن به صورت half-step خواهد بود.

همچنین سطح اتکایی که فرد روی آن خواهد ایستاد ابتدا سفت و سپس بی‌ثبات خواهد شد. برای این کار از فوم، بالشتک‌های تعادلی، rocker board و wobble board استفاده خواهد شد. همچنین مرکز ثقل با اعمال اغتشاش و یا شیفت وزن بوسیله باندهای الاستیک دچار چالش خواهد شد و فرد بایستی ثبات را حفظ کند. این وضعیت‌ها باعث برانگیختن واکنش‌های پوسچرال اتوماتیک و رفلکسی خواهند شد.

مرحله داینامیک

وقتی فرد بتواند ثبات لگن در مرحله قبل را حفظ کند، وارد مرحله داینامیک خواهد شد که در آن فرد حرکات اندام فوقانی و تحتانی را ضمن حفظ ثبات لگن انجام خواهد داد. نحوه‌ی پیشروی در این مرحله مانند مرحله استاتیک از ایستادن روی دو پا، ایستادن بر روی تک پا و سپس ایستادن به صورت half-step خواهد بود. همچنین سطح اتکایی که فرد روی آن خواهد ایستاد ابتدا سفت و سپس بی‌ثبات خواهد شد. در مرحله بعد مرکز ثقل با کمک باندهای الاستیک و پرتاب توپ دچار چالش خواهد شد. این تمرینات باعث بازآموزی مکانیسم‌های فیدفوروارد خواهند شد.

به طور کلی پیشروی تمرینات براساس توانایی هر فرد خواهد بود و هر مرحله تا زمانی انجام خواهد شد که تراپیست به این نتیجه برسد که بیمار ثبات لازم را پیدا کرده و آمادگی انجام تمرینات مرحله بعد را دارد. در این تمرینات مبنا کمیت تمرینات نخواهد بود بلکه کیفیت انجام تمرینات حائز اهمیت خواهد بود.

جدول 2. تمرینات حسی-حرکتی در دو سطح استاتیک و داینامیک

| مراحل | وضعیت | سطح اتکا | چالش جابجایی مرکز ثقل |
| --- | --- | --- | --- |
| استاتیک  حفظ ثبات لگن در شرایط مختلف | ایستادن روی دو پا | سفت | شیفت وزن با باندهای الاستیک و اغتشاش |
|  | ایستادن روی تک پا | فوم |  |
|  | ایستادن در وضعیت half-step | Rocker board |  |
|  |  | Wobble board |  |
| داینامیک  حفظ ثبات لگن در شرایط مختلف ضمن انجام حرکات اندام فوقانی و تحتانی | ایستادن روی دو پا | سفت | شیفت وزن با باندهای الاستیک، اغتشاش و پرتاپ توپ |
|  | ایستادن روی تک پا | فوم |  |
|  | ایستادن در وضعیت half-step | Rocker board |  |
|  |  | Wobble board |  |

#### 17-2-4. تحریک الکتریکی فراجمجمه ای

جهت اعمال جریان الکتریکی از دستگاه tDCS مدل neurostim2 شرکت مدینا طب گستر استفاده خواهد شد. پیش از رسیدن بیمار، تمامی ابزارها شامل الکترودها، نرمال سالین، استیمولاتور، کابل، باندهای الاستیک و تیپ اندازه‌گیری بررسی خواهند شد تا از سالم بودن و عدم وجود خرابی‌ها و آسیب‌های احتمالی اطمینان حاصل شود. بیمار روی صندلی نشسته و پوست سر از نظر وجود هرگونه ضایعه مورد بررسی قرار خواهد گرفت. محقق از شرکت‌کننده درخواست خواهد کرد تا هرگونه تحریک پوستی که در جلسه قبل رخ داده یا هر آنچه که جز معیارهای خروج مطالعه است را گزارش کند. ابتدا الکترودها درون اسفنج آغشته با نرمال سالین قرار داده خواهند شد و پوست محل تحریک با الکل تمیز خواهد شد. سایز الکترودها 2*4 خواهد بود. تحریک از طریق دو الکترود فعال (آنودال) که روی پوست سر قرار خواهند گرفت، اعمال خواهد شد. یک الکترود فعال بر روی منطقه M1 که بر اساس سیستم بین‌المللی 20-10 مطابق با C3 و یا C4 است، قرار خواهد گرفت. الکترود فعال دیگر بر روی منطقه S1 که 2 سانتی‌متر عقب‌تر نسبت به C3 و یا C4 است، قرار خواهد گرفت. الکترودهای رفرنس (کاتدال) نیز روی پیشانی و مستقیماً بالای ابرو قرار خواهند گرفت (تصویر 4). به طور کلی، الکترودهای فعال در سمت مخالف ناحیه درد در کمر و الکترود رفرنس بر روی ناحیه سوپرااوربیتال همان سمت درد جایگذاری خواهند شد. دستگاه روی شدت 5/1 میلی‌آمپر و به مدت 20 دقیقه تنظیم شده و جریان اعمال خواهد شد. در ابتدای شروع جریان، یک دوره‌ی ramping up 10 ثانیه‌ای وجود خواهد داشت که جریان به تدریج به 5/1 میلی‌آمپر خواهد رسید. در انتها نیز یک دوره 10 ثانیه‌ای ramping down وجود خواهد داشت که به تدریج جریان کم شده و دستگاه خاموش خواهد شد. به طور کلی، با توجه به اندازه الکترود فعال، متوسط چگالی جریان زیر این الکترودها 188/0 میلی‌آمپر بر سانتی‌متر مربع خواهد بود. به شرکت‌کننده اطلاع داده خواهد شد که یک احساس قلقلک یا خارشی را تجربه می‌کند و به طور مداوم بیمار در طول درمان تحت نظر قرار خواهد گرفت. در گروه شم نیز الکترودها همانند گروه مداخله جایگذاری خواهند شد. دستگاه روشن شده و فقط جریان طی 10 ثانیه برقرار خواهد بود که شرکت‌کننده احساس گزگز شدن خواهد داشت و بعد از آن جریان قطع خواهد شد. در گروه شم نیز مدت زمان اعمال جریان 20 دقیقه خواهد بود.(32).


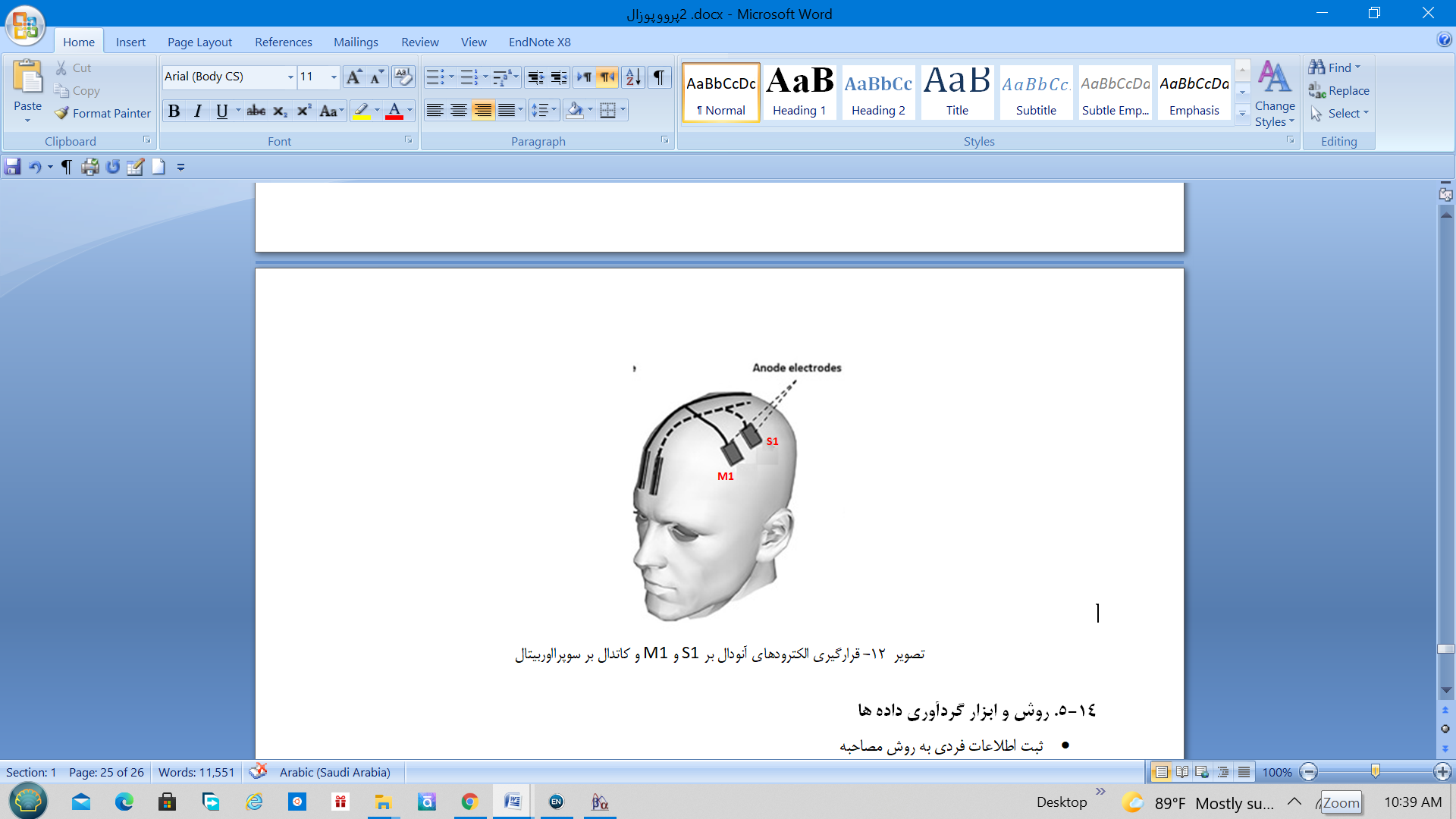


تصویر 4. قرارگیری الکترودهای آنودال بر S1 و M1 و کاتدال بر سوپرااوربیتال

**M1**

## 18. روش تجزیه و تحلیل داده ها

- کلیه پردازش توصیفی و تحلیلی آماری با استفاده از نرم افزار SPSS نسخه 23 صورت میگیرد.
- آمار توصیفی شامل محاسبه شاخص های تمایل مرکزی (میانگین) و پراکندگی (انحراف معیار) است.
- برای ارزیابی توزیع نرمال داده ها از آزمون Shapiro-Wilk استفاده خواهد شد.
- برای مقایسه متغیرهای زمینه ای و متغیرهای وابسته قبل از درمان (پایه) بین دو گروه درمانی از آزمونIndependent t test با سطح معناداری 05/0 استفاده خواهد شد.
- نتایج مربوط به بررسی اثرات عامل درون گروهی مرحله زمانی (قبل و بعد از درمان) و بین گروهی نوع درمان (تمرینات حسی حرکتی با تحریک الکتریکی فراجمجمه ای واقعی و تمرینات حسی حرکتی با تحریک الکتریکی فراجمجمه ای شم) از طریق آزمون Two-Way Mixed between - within ANOVA استفاده خواهد شد. همچنین برای اندازه اثر از معیار **η^2^** استفاده شده و بدین ترتیب (کوچک (01/0)، متوسط (06/0) و بزرگ (14/0)) تفسیر خواهد شد.
- بررسی همبستگی بین پارامترهای بالینی و پارامترهای نوروفیزیولوژیک مربوط به تحریک پذیری حسی و حرکتی کورتکس با استفاده از آزمون Pearson Correlation

## 19. ملاحظات اخلاقی

این مطالعه به تایید کمیته اخلاق دانشگاه علوم پزشکی تهران خواهد رسید و کد اخلاق دریافت میکند. قبل از اجرای آزمون، کلیه مراحل آنبه طور شفاف، روش و هدف انجام آن برای شرکت کنندگان شرح داده میشود و هر یک از افراد شرکت کننده با تکمیل فرم رضایت نامه و به طور آگاهانه در مطالعه شرکت خواهند کرد و در هر مرحله از آزمون در صورت عدم تمایل به همکاری، می توانند از مطالعه خارج شوند. به شرکت کنندگان اطمینان لازم داده میشود که اجرای روند ارزیابی هیچ گونه هزینه ای برای آنها نخواهد داشت و در هنگام انجام ارزیابی ها مراقبتهای لازم برای جلوگیری از هر گونه آسیب به شرکت کنندگان، به عمل خواهد آمد. اطلاعات اخذ شده از افراد فقط جهت تجزیه و تحلیل آماری استفاده خواهد شد و اصل رازداری در این خصوص رعایت میشود. نتایج کلی این مطالعه به صورت گروهی و بدون ذکر هویت افراد گزارش میگردد. هم چنین ابزارهای مورد استفاده در این پژوهش به صورت غیرتهاجمی بوده و هیچ آسیبی به افراد شرکت کننده وارد خواهد نشد.یک مطالعه اخیر در مورد امن بودن tDCS نشان داده است که در 1000 فرد بعد از 33000 جلسه تحریک tDCS، هیچ شواهدی مبنی بر آسیب در این افراد هنگام تست با این پارامتر ها وجود ندارد (≤ 40 min, ≤ 4 mA, ≤ 7.2 C)(55).

1. Affective [↑](#footnote-ref-2)
2. Reorganization [↑](#footnote-ref-3)
3. Adaptive [↑](#footnote-ref-4)
4. Body perception [↑](#footnote-ref-5)
5. Body image [↑](#footnote-ref-6)
6. Excitability [↑](#footnote-ref-7)
7. Sensorimotor integration [↑](#footnote-ref-8)
8. Motivational [↑](#footnote-ref-9)
9. Cognitive [↑](#footnote-ref-10)
10. Dorsolateral prefrontal cortex [↑](#footnote-ref-11)
11. GABA [↑](#footnote-ref-12)
12. Differential control [↑](#footnote-ref-13)
13. Provocative [↑](#footnote-ref-14)
14. Anticipatory [↑](#footnote-ref-15)
15. Sensory motor training [↑](#footnote-ref-16)
16. Transcranial direct current stimulation (tDCS) [↑](#footnote-ref-17)
17. Feasibility [↑](#footnote-ref-18)
18. Effectiveness [↑](#footnote-ref-19)
19. Anticipatory postural adjustments (APA) [↑](#footnote-ref-20)
20. Onset time [↑](#footnote-ref-21)
21. Transversus Abdominis [↑](#footnote-ref-22)
22. External Oblique [↑](#footnote-ref-23)
23. Readiness potential (RP) [↑](#footnote-ref-24)
24. Motor potential (MP) [↑](#footnote-ref-25)
25. Movement-monitoring potential (MMP) [↑](#footnote-ref-26)
26. Visuomotor [↑](#footnote-ref-27)
27. Luomajoki [↑](#footnote-ref-28)
28. Core stability exercises [↑](#footnote-ref-29)
29. Graded [↑](#footnote-ref-30)
30. Pain interference [↑](#footnote-ref-31)
31. Safety [↑](#footnote-ref-32)
