# Supplementary material for: Effects of combining sensory-motor exercises with transcranial direct current stimulation on cortical processing and clinical symptoms in patients with lumbosacral radiculopathy: An exploratory randomized controlled trial
Source: PLoS One. 2024 Dec 19;19(12):e0314361. doi: 10.1371/journal.pone.0314361 (PMC11658635; doi:10.1371/journal.pone.0314361)
Supplement: Attachment — Submitted filename: PONE-D-24-30889.docx [file pone.0314361.s004.docx]

**PONE-D-24-30889 :** Effects of combining sensory-motor exercises with transcranial direct current stimulation on cortical processing and clinical symptoms in patients with chronic low back pain: A sensory and motor-evoked potential study

General comments:

This randomised controlled trial (RCT) by Qanbari et al. tested the effect of combining tDCS with sensorimotor exercises in participants with lumbar radiculopathy compared to sensorimotor exercises alone on pain, disability, motor control and M1/S1 excitability. They observed that the combination of tDCS and sensorimotor exercises did not influence pain and disability more than sensorimotor exercises. Nonetheless, some neurophysiological variables (such as the motor threshold and SEPs amplitude) can be influenced more by tDCS and exercises than exercises alone. They also observed some correlations between changes in motor control and changes neurophysiological variables. Generally, I appreciate reading this study. Here are some methodological issues that need consideration:

- This study should be referred as an exploratory clinical trial. The sample size is small which limits the interpretation of the results that can be done.
- Please change the title to indicate that the population recruited are patients with lumbar radiculopathy instead of low back pain, and change « a sensory and motor-evoked study” for “an exploratory randomised controlled trial”.
- Authors used a per protocol analyses. This type of analysis increases the likelihood to observed differences between groups. Although it is correct to use such analysis to provide real effect of an intervention, providing the results of an intention-to-treat analyses (e.g. by using linear mixed model) can provide data more in line with the reality. An intention-to-treat analysis will include in the analysis all the participants who have been randomised in a study and all participants will be analysed in their allocated group.
- Authors used Pearson’s correlations without testing the assumptions for using parametric testing. Outliers can largely influence the results of parametric testing and I am concerned that the significant correlation between change in AMT and change in motor control is driven by two participants. I would suggest to provide the information about the normality of the dataset and the presence of outliers or the use Spearman’s correlation. Also, for transparency, I encourage the authors the mention the number of correlations that were tested. Considering that they mentioned that the 3 clinical variables were correlated with the 6 neurophysiological variables, I expect that 18 correlation analyses were computed. I suggest to add a Table with the results (r and p values) for all these analyses. I would be also interesting to identify which the group of each data point in the graph (e.g. using different colours) and adding the 95% confidence interval lines.

**Specific comments:**

Introduction

p. 3; ln 72-75: I would nuance a bit this sentence. There is no evidence that low back pain is purely neurophysiological either. It is probably a combination of the different factors. In addition, the reference is a narrative review. I suggest citing some original papers suggesting alteration in CNS.

p. 3-4; ln 87-94: About this section, although Corti et al. demonstrated changes in M1 excitability, TMS targeted M1 area of finger muscles which differs of the rationale presented by the authors(Corti et al., 2022). Thus, I would remove this reference. Also, Tsao et al. (2011) should be cited because they were the first to smudging of M1 maps of the erector spinae in LBP (Tsao et al., 2011). Finally, a recent paper showed that sensorimotor integration was altered in CLBP i.e. that the sensory afferents from the low back area increased corticospinal excitability compared to pain-free controls (Masse-Alarie et al., 2024). This study may help to relate the S1 and M1 paragraphs of the introduction.

p.4; ln 95-98: I would be careful here. The evidence except the one from Jenkins et al. are all case-control studies. I would rather mention that it is possible that some alterations in sensorimotor control may contribute to LBP.

p. 4; ln 104: change « progression » for « maintenance ».

p. 4; ln 111-114 : Other research also demonstrated that motor control exercise can modify the plasticity of the M1, I invite the authors to refer to them (Massé-Alarie et al., 2016, Tsao et al., 2010).

p.4; ln 115: Again, I would change “CLBP is associated” by “some patients with LBP present with a different organization of S1 and M1 areas […]”

p. 5; ln 125: Please refer to systematic review done in LBP (Alwardat et al., 2020, Patricio et al., 2021) that is more related to the topic of the paper.

p. 5; ln 137: change “neuroimaging” for “neurostimulation” if it refers to TMS mapping.

p. 5; ln 139: remove “improved”

**Methods**

p. 6; 162-164: Why did the authors choose to recruit participants with lumbar radiculopathy? Most results in the introduction comes from non-specific LBP. It is less clear if patients with radiculopathy presents changes in M1 and S1. I would be important to add in the introduction some lines on the absence of data in radiculopathy. Also, it would be important to refer to an rTMS and tDCS study who did test its effectiveness in participants with radiculopathy (Attal et al., 2016)

p. 6; ln 174: change “L5/L4 » for “L4/L5”.

p. 6; ln 186-189: These are not exclusion criteria because participants were first included then excluded. These criteria should be rather mentioned in the statistical analysis to describe the per protocol criteria (i.e. the criteria that were used to include a participant in the analysis).

p. 7; ln 192-193: Please describe the mean difference and the standard deviation used to calcite the effect size.

p. 7; ln 207-209: Please indicate the timeframe on which pain was measured (current pain, average pain in the last 24 hours, average pain in the last week, worst pain, etc.). If authors assess current pain, please describe the position of the participants when pain was questioned since it may influence the pain intensity.

p. 9; ln 267-269: For trunk muscles, it is very difficult to obtain MEPs of large amplitude. Most published papers report MEPs amplitude below 100 μV that may make impossible to use this as a criterion for motor threshold (e.g. (Desmons et al., 2024, Desmons et al., 2021)). Did it happen to the authors? Were they able to find MEps over 100 μV in most patients?

p. 10; ln 298-301: Please confirm and add to this section that the intervention lasted for 4 weeks.

p. 11; exercises: What is the rationale to train the foot, lumbar and cervical spine? Why did authors emphasize on these body areas while they were testing only M1/S1 excitability of the low back muscles?

p. 12; ln 364-366: Were assumptions for the use of parametric tests done? With a small sample size, outliers can influence a lot the results of Pearson’s correlations.

**Results**

Table 2: The way the data are presented makes difficult to compare the effects of each intervention. I would suggest presenting the pre-post data for each group side-by-side followed by the post-to-pre mean difference (within-group) with standard deviation or 95% confidence interval. Also, it would be interesting to indicate where there is a significant interaction.

| Experimental | | | Control | | |
| --- | --- | --- | --- | --- | --- |
| Pre | Post | Mean difference | Pre | Post | Mean difference |

p. 13; Ln 390-392: Please indicate if there is any difference between groups for pre and post timepoints each time an interaction occurs to determine if the interaction was explained by differences at baseline or at the follow-up timepoints.

p. 14; linear relationships: Please indicate how many correlations were tested. Please indicate that no other correlation was significant. Did any corrections were applied for multiple testing? Is there any significant correlation between change in motor control and change in pain or disability?

**Discussion**

p. 16; ln 493 change “addressed” for “tested”.

p. 18; Limitations : Please add that the effect of tDCS and exercises were tested on many outcomes. Also, a lot of correlations were tested. This increases the number of type I error. In addition, the sample size was very small.

Alwardat M, Pisani A, Etoom M, Carpenedo R, Chinè E, Dauri M, et al. Is transcranial direct current stimulation (tDCS) effective for chronic low back pain? A systematic review and meta-analysis. Journal of Neural Transmission 2020:1-14.

Attal N, Ayache SS, Ciampi De Andrade D, Mhalla A, Baudic S, Jazat F, et al. Repetitive transcranial magnetic stimulation and transcranial direct-current stimulation in neuropathic pain due to radiculopathy: a randomized sham-controlled comparative study. Pain 2016;157(6):1224-31.

Corti EJ, Marinovic W, Nguyen AT, Gasson N, Loftus AM. Motor cortex excitability in chronic low back pain. Exp Brain Res 2022;240(12):3249-57.

Desmons M, Cherif A, Rohel A, de Oliveira FCL, Mercier C, Masse-Alarie H. Corticomotor Control of Lumbar Erector Spinae in Postural and Voluntary Tasks: The Influence of Transcranial Magnetic Stimulation Current Direction. eNeuro 2024;11(2).

Desmons M, Rohel A, Desgagnes A, Mercier C, Masse-Alarie H. Influence of different transcranial magnetic stimulation current directions on the corticomotor control of lumbar erector spinae muscles during a static task. J Neurophysiol 2021;126(4):1276-88.

Massé-Alarie H, Beaulieu L, Preuss R, Schneider C. Influence of paravertebral muscles training on brain plasticity and postural control in chronic low back pain. Scandinavian Journal of Pain 2016;12:74-83.

Masse-Alarie H, Shraim M, Hodges PW. Sensorimotor Integration in Chronic Low Back Pain. Neuroscience 2024;552:29-38.

Patricio P, Roy JS, Rohel A, Gariepy C, Emond C, Hamel E, et al. The Effect of Noninvasive Brain Stimulation to Reduce Nonspecific Low Back Pain: A Systematic Review and Meta-analysis. Clin J Pain 2021;37(6):475-85.

Tsao H, Danneels LA, Hodges PW. ISSLS prize winner: Smudging the motor brain in young adults with recurrent low back pain. Spine (Phila Pa 1976) 2011;36(21):1721-7.

Tsao H, Galea MP, Hodges PW. Driving plasticity in the motor cortex in recurrent low back pain. Eur J Pain 2010;14(8):832-9.
