# Supplementary material for: Effects of combining sensory-motor exercises with transcranial direct current stimulation on cortical processing and clinical symptoms in patients with lumbosacral radiculopathy: An exploratory randomized controlled trial
Source: PLoS One. 2024 Dec 19;19(12):e0314361. doi: 10.1371/journal.pone.0314361 (PMC11658635; doi:10.1371/journal.pone.0314361)
Supplement: Attachment — Submitted filename: Dear Reviewer 2.docx [file pone.0314361.s005.docx]

**Dear Reviewer 2**

We sincerely appreciate your thorough review of the manuscript and the valuable comments and suggestions provided. We believe that the manuscript has significantly improved as a result of incorporating your feedback, which has been highlighted in blue throughout the text.

**Question 1:** This study should be referred as an exploratory clinical trial. The sample size is small which limits the interpretation of the results that can be done. Please change the title to indicate that the population recruited are patients with lumbar radiculopathy instead of low back pain, and change « a sensory and motor-evoked study” for “an exploratory randomised controlled trial”.

**Answer 1:** We changed the title to “Effects of combining sensory-motor exercises with transcranial direct current stimulation on cortical processing and clinical symptoms in patients with lumbar radiculopathy: an exploratory randomised controlled trial”

**Question 2:** Authors used a per protocol analyses. This type of analysis increases the likelihood to observed differences between groups. Although it is correct to use such analysis to provide real effect of an intervention, providing the results of an intention-to-treat analyses (e.g. by using linear mixed model) can provide data more in line with the reality. An intention-to-treat analysis will include in the analysis all the participants who have been randomised in a study and all participants will be analysed in their allocated group.

**Answer 2:** We revised our analysis and utilized a linear mixed model instead of two-way mixed repeated measures ANOVA. Consequently, we added the following sentences to the statistical analysis section. Although the tables were modified, the final results did not significantly change, so the discussion remains the same.

“To examine the main effects of group (sensory-motor exercises + tDCS vs. sensory-motor exercises + sham tDCS), time (pre- and post-treatment), and their interactions, a linear mixed effects model (LMM) was performed. When significant interactions were identified, the groups were analyzed separately for more detailed insights. Time, group, and their interaction effects were considered fixed effects, while participants were treated as random effects. Mixed models are generally favored over traditional general linear models for handling missing data, as they can utilize all data points, even when some observations are absent. This model evaluates changes over time using maximum likelihood estimation, thus applying an intention-to-treat approach that yields data more reflective of real-world conditions.”

**Question 3:** Authors used Pearson’s correlations without testing the assumptions for using parametric testing. Outliers can largely influence the results of parametric testing and I am concerned that the significant correlation between change in AMT and change in motor control is driven by two participants. I would suggest to provide the information about the normality of the dataset and the presence of outliers or the use Spearman’s correlation. Also, for transparency, I encourage the authors the mention the number of correlations that were tested. Considering that they mentioned that the 3 clinical variables were correlated with the 6 neurophysiological variables, I expect that 18 correlation analyses were computed. I suggest to add a Table with the results (r and p values) for all these analyses. I would be also interesting to identify which the group of each data point in the graph (e.g. using different colours) and adding the 95% confidence interval lines.

**Answer 3:** We revised this section accordingly and included a table that outlines all 18 correlations between clinical and neurophysiological parameters. Moreover, we included 95% confidence interval lines in Figure 4 and distinguished each group in the graph by using different colors.

“Additionally, to examine the relationship between treatment-induced changes (post-test minus pre-test) in neurophysiological and clinical outcomes, Spearman's correlation coefficient was employed, as the change values did not follow a normal distribution. These correlations were computed for all participants regardless of their group assignment. Given that the analysis involved 3 clinical parameters (pain intensity, disability level, and lumbar motor control ability) and 6 neurophysiological parameters (N80 and N150 amplitudes, MEP amplitude, and AMT for multifidus and transversus abdominis/internal oblique muscles), a total of 18 correlations (3×6) were tested. To account for the risk of Type I errors from multiple comparisons, the Bonferroni correction was applied, resulting in a corrected p-value threshold of 0.003.”

Your concern about the relationship between change in AMT and change in motor control was valid, as the revised analysis resulted in a weaker relationship that was no longer statistically significant. We appreciate your careful attention to this matter.

**Introduction**

**Question 4:** p. 3; ln 72-75: I would nuance a bit this sentence. There is no evidence that low back pain is purely neurophysiological either. It is probably a combination of the different factors. In addition, the reference is a narrative review. I suggest citing some original papers suggesting alteration in CNS.

**Answer 4:** We revised this sentence accordingly and included several original studies to support the claim.

“Indeed, the underlying mechanisms of musculoskeletal disorders are not limited to structural and biomechanical factors; neurophysiological processes also play a significant role in their development and progression [6-9].”

**Question 5:** p. 3-4; ln 87-94: About this section, although Corti et al. demonstrated changes in M1 excitability, TMS targeted M1 area of finger muscles which differs of the rationale presented by the authors(Corti et al., 2022). Thus, I would remove this reference. Also, Tsao et al. (2011) should be cited because they were the first to smudging of M1 maps of the erector spinae in LBP (Tsao et al., 2011). Finally, a recent paper showed that sensorimotor integration was altered in CLBP i.e. that the sensory afferents from the low back area increased corticospinal excitability compared to pain-free controls (Masse-Alarie et al., 2024). This study may help to relate the S1 and M1 paragraphs of the introduction.

**Answer 5:** We have removed the Corti et al. study from the references and included the Tsao et al. study. Additionally, we incorporated sentences detailing the findings of the Masse-Alarie et al. study as follow:

“Moreover, a recent study by Masse-Alarie et al. found that sensorimotor integration was disrupted in individuals with CLBP. Specifically, sensory input from the lower back region enhanced corticospinal excitability compared to those without pain. As a result, they recommended interventions aimed at improving both sensory processing and spinal motor control [17].”

**Question 6:** p.4; ln 95-98: I would be careful here. The evidence except the one from Jenkins et al. are all case-control studies. I would rather mention that it is possible that some alterations in sensorimotor control may contribute to LBP.

**Answer 6:** Based on your feedback, we revised these sentences to “These findings indicate that changes in sensorimotor integration and decreased excitability in S1 and M1, which disrupt sensorimotor control, may contribute to chronic and recurrent low back pain.”

**Question 7:** p. 4; ln 104: change « progression » for « maintenance ».

**Answer 7:** We revised it.

**Question 8:** p. 4; ln 111-114 : Other research also demonstrated that motor control exercise can modify the plasticity of the M1, I invite the authors to refer to them (Massé-Alarie et al., 2016, Tsao et al., 2010).

**Answer 8:** We included the two mentioned studies as references.

**Question 9:** p.4; ln 115: Again, I would change “CLBP is associated” by “some patients with LBP present with a different organization of S1 and M1 areas […]”

**Answer 9:** We applied your comment in the text.

**Question 10:** p. 5; ln 125: Please refer to systematic review done in LBP (Alwardat et al., 2020, Patricio et al., 2021) that is more related to the topic of the paper.

**Answer 10:** We included these references to the text.

**Question 11:** p. 5; ln 137: change “neuroimaging” for “neurostimulation” if it refers to TMS mapping.

**Answer 11:** We modified it.

**Question 12:** p. 5; ln 139: remove “improved”

**Answer 12:** We removed “improved”.

Method

**Question 13:** p. 6; 162-164: Why did the authors choose to recruit participants with lumbar radiculopathy? Most results in the introduction comes from non-specific LBP. It is less clear if patients with radiculopathy presents changes in M1 and S1. I would be important to add in the introduction some lines on the absence of data in radiculopathy. Also, it would be important to refer to an rTMS and tDCS study who did test its effectiveness in participants with radiculopathy (Attal et al., 2016)

**Answer 13**: Based on your feedback, we included the following paragraphs to the introduction

“Chronic low back pain (CLBP) is one of the most prevalent health issues worldwide, affecting up to 80% of individuals, with 5% to 10% suffering lumbosacral radiculopathy [1]. This condition is characterized by radicular pain resulting from the compression or irritation of nerve roots in the lumbosacral region of the spine [2]. CLBP is a multifaceted sensory and emotional condition influenced by mechanical, psychological, and psychosocial factors [3]. Consequently, many patients experience persistent radicular symptoms, and some may require surgical intervention. However, not all patients achieve satisfactory pain relief, even with appropriate treatments [4]. Thus, the underlying cause of persistent pain remains unclear. Evidence indicates that treatments focused solely on correcting structural abnormalities in the musculoskeletal system are often unsuccessful, suggesting the involvement of additional pathophysiological and biopsychosocial mechanisms [5].”

“It is important to note that most of these findings are derived from non-specific CLBP, while research on changes in the M1 and S1 in patients with lumbosacral radiculopathy is limited. Nevertheless, studies have indicated that brain networks are also affected in these patients [4, 19].”

Additionally, a study by Attal et al. on patients with lumbosacral radiculopathy found that repetitive transcranial magnetic stimulation (rTMS) over M1 was more effective than tDCS and sham treatments, potentially modulating both the sensory and affective aspects of pain [34].

**Question 14:** p. 6; ln 174: change “L5/L4 » for “L4/L5”.

**Answer 14:** We corrected it.

**Question 15:** p. 6; ln 186-189: These are not exclusion criteria because participants were first included then excluded. These criteria should be rather mentioned in the statistical analysis to describe the per protocol criteria (i.e. the criteria that were used to include a participant in the analysis).

**Answer 15**: We removed this section because, as you suggested, the intention-to-treat approach was utilized. Including these cases could potentially confuse readers regarding whether all participants were ultimately included in the analysis or if some left and were excluded.

**Question 16:** p. 7; ln 192-193: Please describe the mean difference and the standard deviation used to calcite the effect size.

**Answer 16**: We added the mean difference and the standard deviation to the text as follow:

“The sample size was determined using G*Power 3.1.9.2 software, following the methodology of the Jafarzadeh et al. study [35], with pain as the primary outcome measure. The calculation utilized a between-group Cohen's d of 1.09, derived from the mean difference of 1.42 divided by the pooled standard deviation of 1.30, with a power of 0.8 and an alpha level of 0.05. This power analysis indicated that a minimum of 30 participants was required. To accommodate a potential dropout rate of 15%, a total of 34 participants were recruited.”

**Question 17:** p. 7; ln 207-209: Please indicate the timeframe on which pain was measured (current pain, average pain in the last 24 hours, average pain in the last week, worst pain, etc.). If authors assess current pain, please describe the position of the participants when pain was questioned since it may influence the pain intensity.

**Answer 17**: We evaluated 'average pain over the past week' and have included this in the text.

**Question 18:** p. 9; ln 267-269: For trunk muscles, it is very difficult to obtain MEPs of large amplitude. Most published papers report MEPs amplitude below 100 μV that may make impossible to use this as a criterion for motor threshold (e.g. (Desmons et al., 2024, Desmons et al., 2021)). Did it happen to the authors? Were they able to find MEps over 100 μV in most patients?

**Answer 18**: We applied stimulation during sub-maximal muscle contraction (15-20% MVC) to achieve better recordings compared to the resting state. The coil type and hotspot were selected based on previous studies for optimal results. For the MEP amplitude, we conducted 10 stimulations at 120% AMT at the hotspot, averaging the 5 highest responses. This led to slightly higher values compared to those in the Desmons et al. study. However, following your recommendation, we reviewed the data and recalculated the average using all 10 stimulations, which brought our results closer to those reported in Desmons et al. Despite this adjustment, there was no significant alteration in the overall analytical pattern. Additionally, for three participants where recording posed challenges, the intensity was raised to as high as 70% of the maximum stimulator output to determine an appropriate AMT.

**Question 19:** p. 10; ln298-301: Please confirm and add to this section that the intervention lasted for 4 weeks.

**Answer 19**: We added the 4-week duration to this section.

**Question 20:** p. 11; exercises: What is the rationale to train the foot, lumbar and cervical spine? Why did authors emphasize on these body areas while they were testing only M1/S1 excitability of the low back muscles?

**Answer 20**: The rationale for incorporating training of the foot, lumbar spine, and cervical spine in sensorimotor training lies in the inherent interconnectedness of the sensory and motor systems, a concept highlighted by Vladimir Janda. These regions are abundant in proprioceptors, which deliver vital sensory feedback to the CNS for maintaining balance and joint stability. By optimizing sensory input from these areas, the training enhances movement patterns, boosts low back muscle activation, and fosters automatic, coordinated movements.

Additionally, proper joint positioning at these critical points is crucial for sustaining a neutral posture; any dysfunction can lead to compensatory movement patterns that negatively impact low back muscle function. This positioning ensures that mechanoreceptors effectively transmit essential postural information to the CNS. Although the study concentrated on M1/S1 excitability of the low back muscles, training the foot, lumbar spine, and cervical spine is integral to improving overall sensorimotor function, which ultimately contributes to enhanced movement patterns and a lower risk of injury.

1. Page PJJob, therapies m. Sensorimotor training: A “global” approach for balance training. 2006;10(1):77-84.

2. Rogers ME, Page P, Takeshima NJIjospt. Balance training for the older athlete. 2013;8(4):517.

**Question 21:** p. 12; ln 364-366: Were assumptions for the use of parametric tests done? With a small sample size, outliers can influence a lot the results of Pearson’s correlations.

**Answer 21**: We reanalyzed the correlation using Spearman's correlation coefficient to account for non-normal distribution in the data.

Results

**Question 22:** Table 2: The way the data are presented makes difficult to compare the effects of each intervention. I would suggest presenting the pre-post data for each group side-by-side followed by the post-to-pre mean difference (within-group) with standard deviation or 95% confidence interval. Also, it would be interesting to indicate where there is a significant interaction.

| Experimental | | | Control | | |
| --- | --- | --- | --- | --- | --- |
| Pre | Post | Mean difference | Pre | Post | Mean difference |

**Answer 22**: We revised Table 2 in accordance with your feedback. Additionally, we highlighted the significant interaction effects, as well as the time and group effects, in Table 3.

**Question 23:** p. 13; Ln 390-392: Please indicate if there is any difference between groups for pre and post time points each time an interaction occurs to determine if the interaction was explained by differences at baseline or at the follow-up time points.

**Answer 23**: We initially stated in the statistical analysis section that there are no differences between the groups before treatment, establishing their comparability. In other instances, including the following, we made an effort to implement your feedback.

“For the AMT of TrA/IO, both the interaction and group effects were significant. After separating the groups and analyzing them individually, it was found that in the intervention group, the AMT significantly decreased from pre-test to post-test, F(1,16.499) = 4.094, P = 0.049, Cohen’s d = 0.997. However, there was no significant change over time in the control group, F(1,15.765) = 1.108, P = 0.308, Cohen’s d = 0.530. Moreover, sensory-motor exercises combined with tDCS demonstrated a significantly lower AMT in the post-test compared to those combined with sham tDCS, F(1,30) = 13.142, P = 0.001, Cohen’s d = 1.324.”

**Question 24:** p. 14; linear relationships: Please indicate how many correlations were tested. Please indicate that no other correlation was significant. Did any corrections were applied for multiple testing? Is there any significant correlation between change in motor control and change in pain or disability?

**Answer 24**: We added the following paragraph to the text and included Table 4, which displays the correlation between all neurophysiological and clinical outcomes. We did not present the correlations within neurophysiological and clinical outcomes together. However, in line with your question, we did not find a significant correlation between changes in motor control and changes in pain or disability.

“Additionally, to examine the relationship between treatment-induced changes (post-test minus pre-test) in neurophysiological and clinical outcomes, Spearman's correlation coefficient was employed, as the change values did not follow a normal distribution. These correlations were computed for all participants regardless of their group assignment. Given that the analysis involved 3 clinical parameters (pain intensity, disability level, and lumbar motor control ability) and 6 neurophysiological parameters (N80 and N150 amplitudes, MEP amplitude, and AMT for multifidus and transversus abdominis/internal oblique muscles), a total of 18 correlations (3×6) were tested. To account for the risk of Type I errors from multiple comparisons, the Bonferroni correction was applied, resulting in a corrected p-value threshold of 0.003.”

**Discussion**

**Question 25:** p. 16; ln 493 change “addressed” for “tested”.

**Answer 25**: We corrected it.

**Question 26:** p. 18; Limitations: Please add that the effect of tDCS and exercises were tested on many outcomes. Also, a lot of correlations were tested. This increases the number of type I error. In addition, the sample size was very small.

**Answer 26**: We incorporated these concerns into the Limitations section.
