# Supplementary material for: Effects of combining sensory-motor exercises with transcranial direct current stimulation on cortical processing and clinical symptoms in patients with lumbosacral radiculopathy: An exploratory randomized controlled trial
Source: PLoS One. 2024 Dec 19;19(12):e0314361. doi: 10.1371/journal.pone.0314361 (PMC11658635; doi:10.1371/journal.pone.0314361)
Supplement: Attachment — Submitted filename: Dear Reviewer 1.docx [file pone.0314361.s006.docx]

**Dear Reviewer 1**

We sincerely appreciate your thorough review of the manuscript and the valuable comments and suggestions provided. We believe that the manuscript has significantly improved as a result of incorporating your feedback, which has been highlighted in red throughout the text.

**Question 1:** 1- Table 1: Check the data for normal distributions. If variables do not follow normal distributions, summarize using median, first and third quartiles and compare using Wilcoxon rank sum test

**Answer 1:** We reviewed and revised them in Table 1. Furthermore, the related text was modified accordingly.

“The Shapiro-Wilk test was used to evaluate the normality of the data distribution. Most data followed a normal distribution, except for certain demographic characteristics like height, weight, and pain duration. The Chi-square test was used to compare the proportions of gender and pain side between the groups. For parameters with a normal distribution, an independent t-test was conducted, while the Wilcoxon rank-sum test was applied to non-normally distributed parameters. The results indicated no significant baseline differences between the two groups, confirming their comparability before the intervention.”

**Question 2:** Lines 250 & 314: The standard statistical term for “average” is “mean”.
**Answer 2:** We revised it.

**Question 3:** Line 401: The chi-square test compares proportions rather than frequencies.

**Answer 3:** We revised it.
